# Liquid–Solid Interface Reactions Drive Enhanced Thermoelectric Performance in Ag_2_Se

**DOI:** 10.1021/jacs.5c11435

**Published:** 2025-08-22

**Authors:** Yu Liu, Tobias Kleinhanns, Sharona Horta, Ewelina P. Dutkiewicz-Kopczynska, Shaoqing Lu, Maria Chiara Spadaro, Aziz Genç, Lei Chen, Khak Ho Lim, Min Hong, Jordi Arbiol, Maria Ibáñez

**Affiliations:** † Anhui Province Engineering Research Center of Flexible and Intelligent Materials, School of Chemistry and Chemical Engineering, 558979 Hefei University of Technology, 230009, Hefei, China; § 148492 Institute of Science and Technology Austria (ISTA), Am Campus 1, 3400, Klosterneuburg, Austria; ¶ Catalan Institute of Nanoscience and Nanotechnology (ICN2), CSIC and BIST, Campus UAB, Bellaterra, 08193, Barcelona, Catalonia Spain; ∥ Centre for Future Materials and School of Engineering, 7932 University of Southern Queensland, Springfield Central Queensland, 4300, Australia; ◊ Institute of Zhejiang University-Quzhou, 78 Jiuhua Boulevard North, Quzhou 324000, Zhejiang China; + ICREA, Pg. Lluís Companys 23, Barcelona, Catalonia 08010, Spain

## Abstract

Ag_2_Se is a promising n-type thermoelectric material, but its performance is limited by excessive carrier concentration, compositional inhomogeneity, and phase instability, challenges rooted in a narrow homogeneity range and uncontrolled Ag^+^ diffusion in the superionic phase. Here, we address these issues by exploiting liquid–solid interface reactions using CdSe complexes that remove surface excess Ag to yield stoichiometric Ag_2_Se and generate CdSe nanodomains that inhibit Ag^+^ diffusion and constrain grain growth. The resulting Ag_2_Se-CdSe nanocomposites exhibit a reproducible, stable figure of merit (*zT*) of 1.04 between 300 and 390 K. Beyond demonstrating high performance, we elucidate the interfacial chemical reactions that give rise to the observed microstructure and transport properties, providing a foundation for rationally engineering interfacial chemistry to tailor transport properties across diverse thermoelectric material systems.

## Graphical abstract

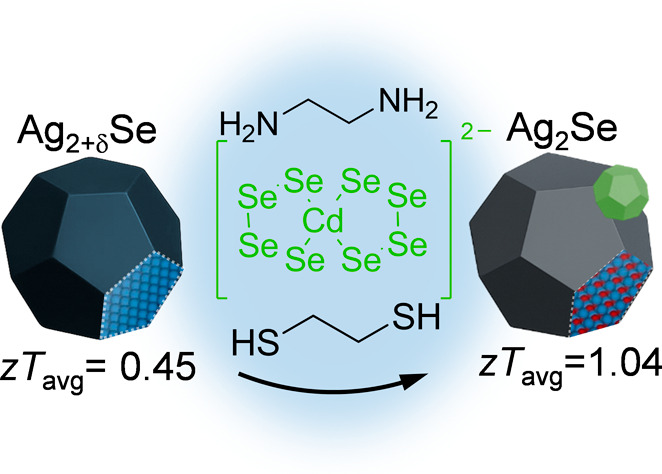

## Introduction

Thermoelectric (TE) materials, which can convert heat into electricity and vice versa, play a pivotal role in advancing technologies such as compact cooling systems in 5G networks, enabling seamless integration into the Internet of Things (IoT), and body heat self-powering technologies. However, the full potential of these materials has yet to be realized, constrained by their less-than-ideal performance, complex manufacturing processes, and challenges associated with integrating them into functional devices.^1–3^ Moreover, there is a need to move beyond the reliance on Bi_2_Te_3_-based compounds, which are the benchmark for n-type TE materials near room temperature. The reasons are the scarcity of tellurium, the complex processing requirements of Bi_2_Te_3_, and its typically poor mechanical properties, which create significant barriers to the widespread application of TE technology.^4–8^

In light of these challenges, Ag_2_Se has emerged as a promising alternative challenging the predominance of Bi_2_Te_3_-based TE materials in the temperature range from room temperature up to approximately 403 K.^9–12^ Above 403 K, Ag_2_Se undergoes a phase transition from the orthorhombic β-Ag_2_Se to the cubic α-Ag_2_Se phase, where Ag ions become highly mobile. Although such liquid-like behavior is beneficial for reducing the lattice thermal conductivity (*κ*_*L*_), the excessively high charge carrier concentration (*n*_*H*_) limits the use of Ag_2_Se as TE to below the phase transition temperature.^13,14^ In this temperature range, the interdependence between charge carrier and phonon transport can be optimized toward a high TE figure of merit, *zT* = *σS* ^2^ *T*/*κ*_*tot*_, where σ is the electrical conductivity, *S* is the Seebeck coefficient and *κ*_*tot*_ is the total thermal conductivity.^15–17^

Efforts to increase the *zT* of β-Ag_2_Se have been made through a range of methodologies, including the incorporation of secondary phases,^18–22^ alloying/doping,^23–28^ defect engineering,^9,14,29–31^ and the precise manipulation of stoichiometry.^32–37^ Despite promising results, large discrepancies in temperature-dependent tendencies, mainly related to stoichiometry and defect control, are hindering the pathway toward consistent performance. This primarily arises from the relatively weak Ag–Se bond ionicity,^12,38^ facilitating the migration of Ag ions to interstitial sites and the volatilization of Se at elevated temperatures, which results in significantly higher *n*_*H*_ than those required for maximum *zT*.^10,12,14,28^ Particularly, synthesizing Ag_2_Se with control over stoichiometry via solid-state methods is challenging, as seen by the considerable variations in the transport parameters in samples with the same nominal composition due to the formation of secondary phases.^10,14,39^ Alternatively, solution-based synthesis can be a powerful strategy to achieve the desired stoichiometry, defect type and defect density, thereby translating particle-level control into a macroscopic solid.^14,15,40^ This is because the liquid-phase medium plays a critical role in enabling selective reaction pathways, minimizing impurity incorporation,^14,40,41^ and supporting dynamic dissolution–precipitation processes through its ability to stabilize dissolved species and mediate reaction kinetics.^42^

Beyond direct synthesis in solution, post-synthetic modifications in a liquid-phase medium enable well-controlled surface reactions,^41,43,44^ allowing the fine-tuning of the powder composition before consolidation into dense pellets, and offering a flexible and effective approach to optimizing material properties. However, research has primarily focused on three objectives: removing native insulating surface ligands from surfactant-assisted synthesis,^45^ eliminating surface oxides through acid treatment,^46^ and functionalizing surfaces with inorganic molecular complexes that, upon thermal decomposition and solid-state reactions during the sintering process, tune composition and defects.^47–49^

Herein, we exploit reactions at the liquid–solid interface to tune the particle composition after synthesis. These post-synthetic reactions enable precise control over both stoichiometry and defect populations in the final dense material, and we reveal the interfacial chemical transformations that govern material evolution. This strategy is particularly critical for Ag_2_Se, whose single-phase stability exists within a very narrow compositional range: even slight surface excess of Ag or Se leads to phase segregation during sintering. Through a solution-based modification protocol, excess Ag is removed to attain stoichiometry while CdSe domains are introduced to form an Ag_2_Se-CdSe nanocomposite (NCP). The resulting NCPs achieve a reproducible *zT* of ≈1.04 from 300 to 390 K. By elucidating the underlying chemistry alongside performance metrics, this work provides a foundation for rational, design-driven control of interfacial reactions.

## Results and Discussion

Ag_2_Se particles are synthesized through an aqueous route using silver nitrate (AgNO_3_) and selenium (Se) powder precursors, following a series of purification steps to remove dissociated ionic species (Supporting Information, SI). Adjusting the Ag^+^:Se^2–^ ratio to 2:1.1 is necessary to produce Ag_2_Se powder with no detectable metallic Ag phase by X-ray diffraction (XRD) (Figure S1A) and X-ray photoemission spectroscopy (XPS) (Figure S2). However, even in the absence of a detectable Ag^0^ phase, elemental analysis indicates that the as-synthesized Ag_2_Se powder is slightly rich in Ag^+^ (Figure S1B). Considering the extremely low solubility of Ag in Ag_2_Se and the relatively large size of the particles (Figure S3A), we believe most of this excess is due to surface atoms. Upon thermal processing and densification using spark plasma sintering (SPS), this surface excess of Ag results in metallic Ag nanodomains in the final composite (discussed later in the Pellets microstructure section), which are well-known to increase the charge carrier concentration and ultimately degrade the TE performance.^36,50^

Due to the limited control over the surface termination through direct powder synthesis, we explore the possibility of removing the excess of Ag through post-synthetic reactions at the liquid–solid interface. To this aim, Ag_2_Se particles are blended with CdSe molecular complexes (Figure S4). After the reaction is completed, the surface-modified Ag_2_Se particles are isolated from the liquid phase, annealed under a nitrogen flow and consolidated into dense pellets by SPS, resulting in Ag_2_Se-x%CdSe (x = 0, 2, 4, 6, 7, and 10) NCPs. All the details of the material synthesis can be found in the Supporting Information (SI).

All produced pellets are highly dense, with relative densities exceeding 95% of the theoretical value (Table S1). The composition of these pellets closely matches the nominal values, confirmed by inductively coupled plasma optical emission spectroscopy (ICP-OES) analysis (Table S2). The crystal structure of both the annealed powders (Figure S5) and the consolidated pellets (Figure 1A-C) corresponds to the orthorhombic β-Ag_2_Se structure with the *P*2_1_2_1_2_1_ space group, and the Rietveld refinement indicates that the surface modification with CdSe molecular complexes does not alter the lattice parameters (Figure S6, Table S3). These findings suggest that Cd^2+^ ions are not substituting cation sites within the orthorhombic lattice of β-Ag_2_Se; otherwise, the significant size difference between Ag^+^ ions (∼1.26 Å) and Cd^2+^ ions (∼0.96 Å) would have led to notable lattice distortions.^51^ Moreover, temperature-dependent XRD studies show that within the studied temperature range CdSe neither forms a solid solution with Ag_2_Se, nor does it influence the phase transition temperature (Figure S7). These results agree with the limited solubility shown in the phase diagram between Ag_2_Se and CdSe below 500 °C.^52^ Therefore, CdSe exists as a secondary phase within the Ag_2_Se matrix throughout the investigated temperature range.

**Figure 1:**
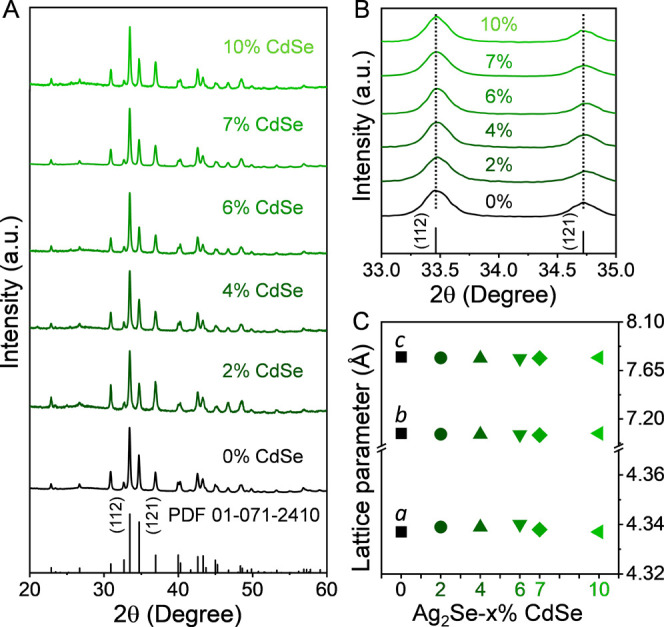
A) XRD patterns of the pellets obtained from Ag_2_Se-x%CdSe (x = 0, 2, 4, 6, 7 and 10) NCPs, including the Ag_2_Se reference pattern (PDF 01-071-2410). B) Magnification of the (112) and (121) XRD peaks at ca. 2θ = 33.5° and 34.7° with dotted vertical lines to guide the eyes. C) Lattice parameters *a*, *b* and *c* calculated from XRD patterns.

### Transport Properties

Given that the material exhibits high TE performance only below the phase transition temperature,^10–13^ we focus primarily on investigating the transport properties of the low-temperature β-Ag_2_Se phase within the 300–390 K temperature range. The transport properties beyond the phase transition are presented in Figure S8.

In this study, the untreated Ag_2_Se sample exhibits a higher σ than most reported bulk Ag_2_Se-based systems, reaching 2.2 × 10^3^ S cm^–1^ at room temperature (Figure 2A).^14,19–21,23,25,34^ Conversely, the σ significantly decreases for all Ag_2_Se-x%CdSe NCPs, with smaller values for those with larger CdSe content. For instance, the room temperature σ of the Ag_2_Se-10%CdSe NCP decreases to 950 S cm^–1^, more than a 2.3-fold reduction compared to the untreated Ag_2_Se sample. Correspondingly, the absolute value of *S* increases with CdSe content, e.g., the *S* value for the Ag_2_Se-7%CdSe sample is −169 μV K^–1^ at room temperature, roughly 1.45 times higher than that of the untreated Ag_2_Se (Figure 2B).

**Figure 2:**
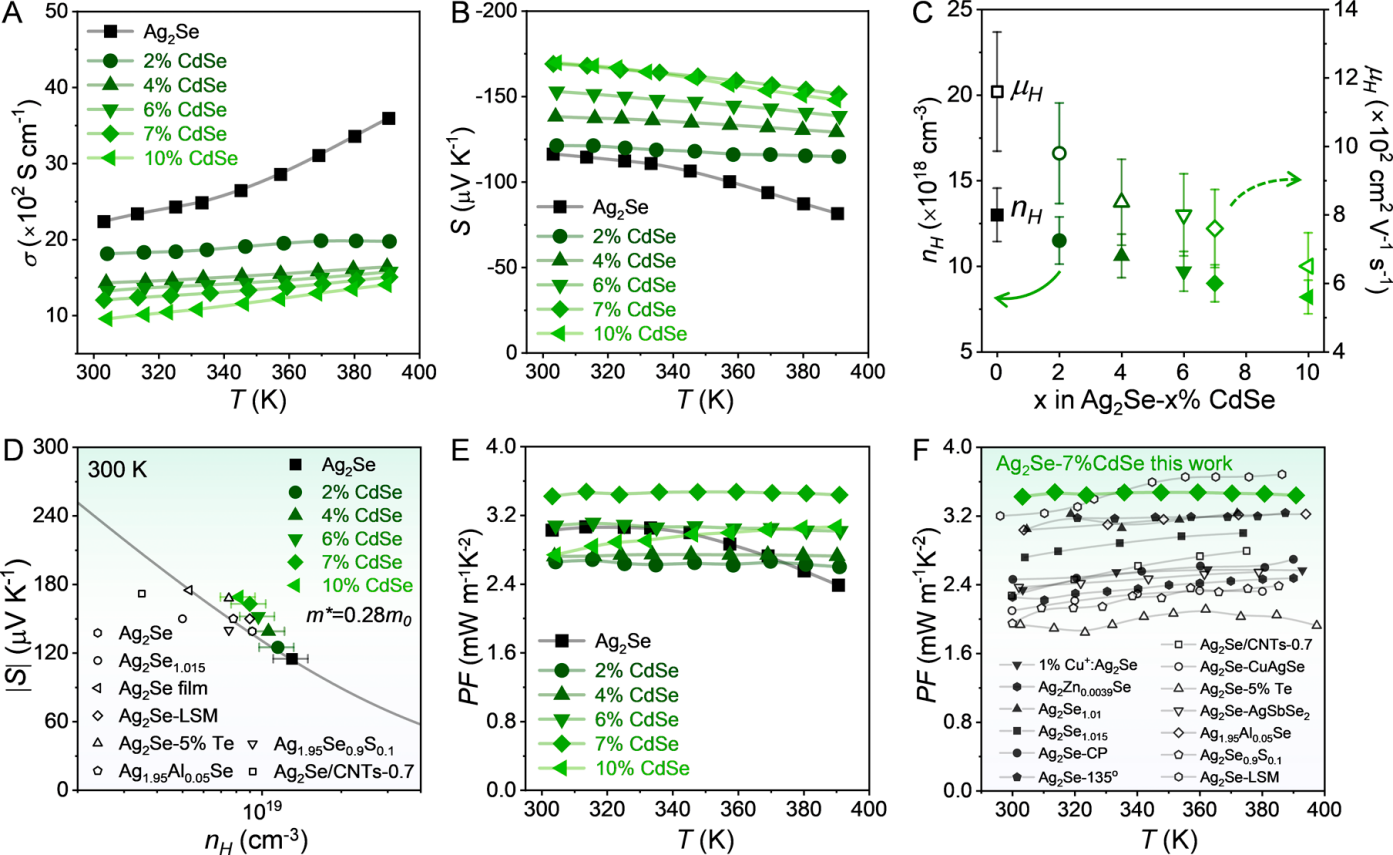
Electrical properties of Ag_2_Se-x%CdSe NCPs (x = 0, 2, 4, 6, 7 and 10). Temperature dependence of A) electrical conductivity (σ), B) Seebeck coefficient (*S*), and C) Hall charge carrier concentration (*n*_*H*_, solid symbols) and carrier mobility (*μ*_*H*_, open symbols) at ambient temperature. D) Pisarenko plot at 300 K. with black open symbols representing reference data from other Ag_2_Se-based TE materials.^18,19,24,25,31,53^ E) Temperature dependence of power factor (*PF*) of these NCPs. F) Comparison of PF values of the Ag_2_Se-7%CdSe sample with those reported state-of-the-art Ag_2_Se-based TE materials.^18–21,23–26,30–34^

To understand the variations in σ and *S* with differing CdSe content, we conducted room temperature Hall measurements to determine the *n*_*H*_ and *μ*_*H*_ (Table S4). Figure 2C shows that the *n*_*H*_ value for the untreated Ag_2_Se is 1.3 × 10^19^ cm^–3^, which is significantly higher than its optimal range ∼10^18^ cm^–3^. All samples containing CdSe exhibit lower *n*_*H*_ values than the untreated Ag_2_Se, progressively decreasing with increasing CdSe content, e.g., in the Ag_2_Se-10%CdSe NCP the *n*_*H*_ is to 8.0 × 10^18^ cm^–3^. All surface-modified samples have lower *μ*_*H*_ values at room temperature compared to the untreated Ag_2_Se, decreasing with rising CdSe content, e.g., *μ*_*H*_ decreased from 1160 cm^2^ V^–1^ s^–1^ in untreated Ag_2_Se to approximately half at 650 cm^2^ V^–1^ s^–1^ in Ag_2_Se-10%CdSe (Figure 2C). The temperature-dependent measurements of *n*_*H*_ (Figure S8C) and *μ*_*H*_ (Figure S8D) for Ag_2_Se and the Ag_2_Se-7%CdSe sample show that *n*_*H*_ increases with rising temperature, but much more significantly for the untreated Ag_2_Se.

The *S* is inversely proportional to the *n*_*H*_, with the samples exhibiting lower carrier densities showing higher absolute values of *S*. We further explored the relationship between *S* and *n*_*H*_ using the Single Parabolic Band (SPB) model and the derived Pisarenko relation, as compared with previously reported data (Figure 2D).^18,19,24,25,31,53^ This analysis shows that the *S* values are relatively higher and increasingly deviate from the theoretical curve as the CdSe content increases. The σ and *S* values for Ag_2_Se-x%CdSe NCPs (x = 0, 2, 4, 6, 7, and 10) were used to calculate the power factor (*PF* = *σS* ^2^), as shown in Figure 2E. Notably, the Ag_2_Se-7%CdSe sample shows one of the highest reported *PF* values across the entire temperature range, reaching ∼3.4 mW m^–1^ K^–2^ at room temperature (Figure 2F).^18–21,23–26,30–34^

Ag_2_Se typically exhibits intrinsically low *κ*_*L*_,^11,54^ where from the phonon dispersion relation (Figure 3A) the low and high-frequency modes are predominantly governed by Ag and Se atoms, respectively (Figure 3B). From the slope of the phonon dispersion curve, the phonon group velocity (υ), is calculated as υ = dω/d*q*, where ω and *q* represent the frequency and wave vector of phonons, respectively. The variation of υ with ω is depicted in Figure 3C, where the group velocity is comparable to that of typical TE materials with inherently low *κ*_*L*_. Additionally to a small group velocity, the hybridization of optical and acoustic branches enhances phonon scattering, thereby contributing to the inherently low *κ*_*L*_ in Ag_2_Se.

**Figure 3:**
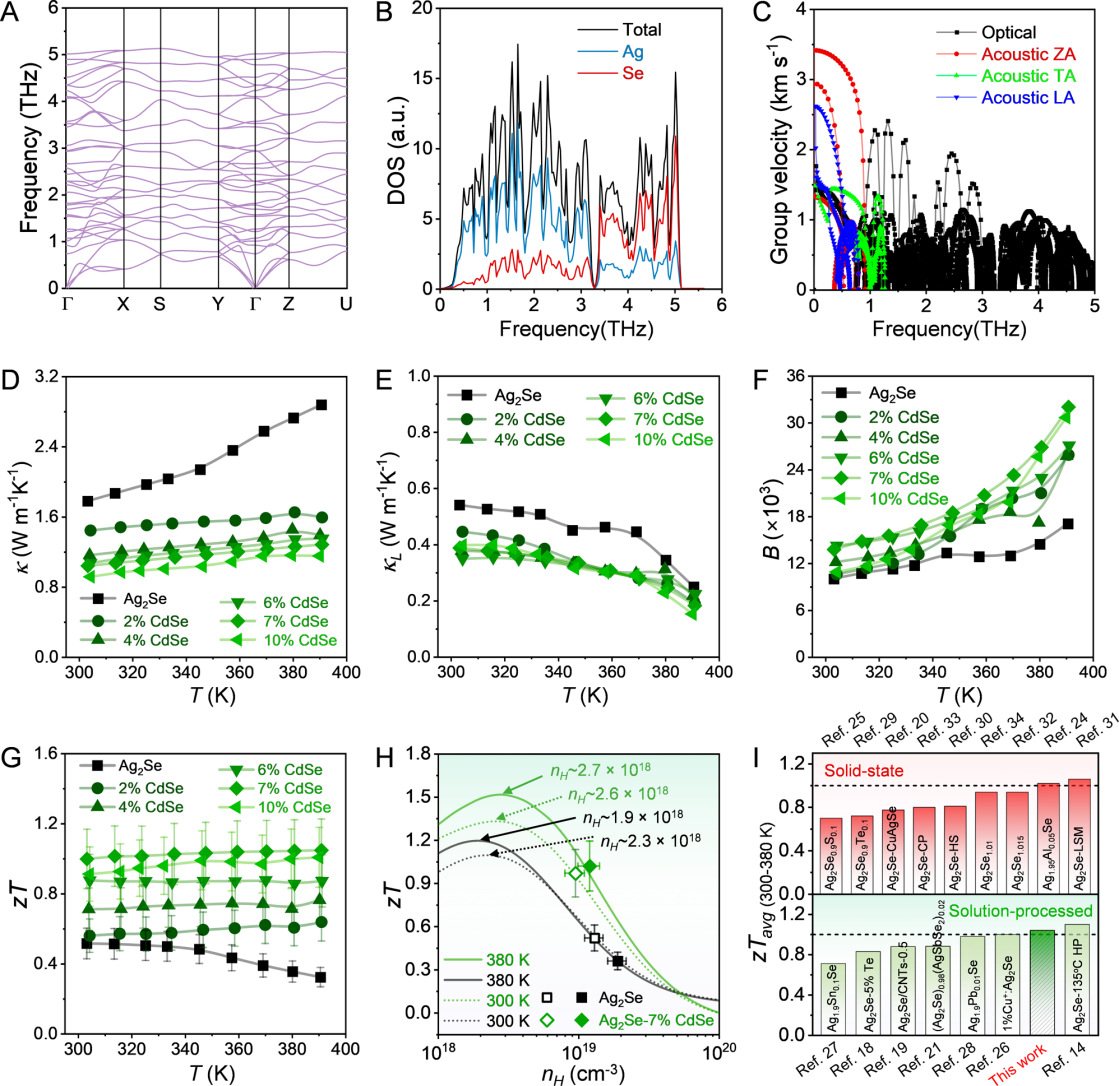
A–C) phonon dispersion, PDOS and phonon group velocity of ZA, TA, LA and optical phonon modes of Ag_2_Se at 300 K, respectively. Temperature dependence of D) thermal conductivity (*κ*_*tot*_), E) lattice thermal conductivity (*κ*_*L*_) for Ag_2_Se-x%CdSe NCPs (x = 0, 2, 4, 6, 7 and 10). F) Quality factor (*B*), G) Temperature dependence of the TE figure of merit (*zT*) for Ag_2_Se-x%CdSe NCPs (x = 0, 2, 4, 6, 7 and 10), H) *n*_*H*_ dependent *zT* with both experimental and predicted values for Ag_2_Se and Ag_2_Se-7%CdSe at *T* = 300 and 380 K via calculations using the SPB model. The arrows indicate the optimal carrier concentration (*n*_*H*_) for peak *zT* values. I) Comparison of *zT*_*avg*_ values of the Ag_2_Se-7%CdSe sample with those of state-of-the-art Ag_2_Se-based TE materials: produced by solid-state technology,^20,24,25,29–34^ and solution-processed technology,^14,18,19,21,26–28^ in the low-temperature range of 300 to 380 K.

The temperature-dependent *κ*_*tot*_ of all samples is shown in Figure 3D. Compared to untreated Ag_2_Se, all Ag_2_Se-CdSe NCPs exhibit much lower *κ*_*tot*_ across the entire measured temperature range. The *κ*_*L*_ and electronic thermal conductivity (*κ*_*e*_) of all samples are calculated according to the Wiedemann–Franz Law, and are presented in Figures 3E and S9, respectively: *κ*_*e*_ = *L*_*o*_ *σT* and *κ*_*L*_ = *κ*_*tot*_ – *κ*_*e*_, where *L*_*o*_ is Lorenz number.^55^ As the CdSe content increases, the *κ*_*e*_ values of the Ag_2_Se-CdSe NCPs gradually decrease, primarily due to the reduction in σ (Figure 2A), contributing to the decrease in *κ*_*tot*_. Moreover, all Ag_2_Se-CdSe samples have lower *κ*_*L*_ values compared to untreated Ag_2_Se. The lowest *κ*_*L*_ is found in Ag_2_Se-10%CdSe and decreases from 0.38 W m^–1^ K^–1^ at room temperature to 0.15 W m^–1^ K^–1^ at 390 K (Figure S9D).

From the ensemble of electronic and thermal transport descriptors, the quality factor (*B*) is directly proportional to *μ*_*W*_/*κ*_*L*_ and indicates the potential for a high *zT*, considering that the material can be optimally doped.^56,57^ In comparison to untreated Ag_2_Se, *B* is notably higher in all NCPs, with Ag_2_Se-7%CdSe exhibiting the highest values across the entire temperature range (Figure 3F).

The TE figure of merit *zT* values of all Ag_2_Se-x%CdSe NCPs (x = 0, 2, 4, 6, 7, and 10) as a function of temperature are shown in Figure 3G. All samples treated with CdSe molecular complexes exhibit higher *zT* values than the untreated Ag_2_Se. The Ag_2_Se-7%CdSe NCPs exhibit the highest relative *PF* and a relatively low *κ*_*tot*_, resulting in a maximum *zT* value of ∼1.05 at 380 K. Furthermore, the *n*_*H*_ of the Ag_2_Se-7%CdSe NCP is higher than that predicted to be optimal by the SPB model (Figure 3H), indicating that reducing *n*_*H*_ to ∼2.6 × 10^18^ cm^–3^ could potentially increase the room temperature *zT* value beyond 1.3. More important for practical applications, the *zT* remains nearly constant throughout the measured temperature range, yielding a high average *zT* value (*zT*_*avg*_) of ∼1.04 for the Ag_2_Se-7%CdSe NCP within the low-temperature range from 300 to 390 K (Figure 3I), using the following formula [Disp-formula eq1]:

1 $$zT_{avg}=\frac{1}{T_{h}-T_{c}}\int_{T_{c}}^{T_{h}}zTdT$$

where *T*_*h*_ and *T*_*c*_ are the temperatures of the hot side and cold side, respectively. This value exceeds those of most Ag_2_Se-based materials produced by both solid-state,^20,24,25,29–34^ and solution-processed methods.^14,18,19,21,26,27^ The TE power generation efficiency (η) of actual devices depends on *zT*_*avg*_, as demonstrated by the eq 2.^17^ The estimated theoretical η for the Ag_2_Se-7%CdSe sample is ∼4.5% at a temperature difference (*T*_*h*_ – *T*_*c*_) of 90 K and a cold side temperature of *T*_*c*_ = 300 K.

2 $$\eta =\frac{T_{h}-T_{c}}{T_{h}}\left[\frac{\sqrt{1+zT_{avg}}-1}{\sqrt{1+zT_{avg}}+\frac{T_{c}}{T_{h}}}\right]$$

The results obtained from Ag_2_Se-CdSe NCPs exhibit remarkable stability, maintaining consistent performance during thermal cycling tests (Figure S10). Moreover, the reproducibility of these results has been confirmed across multiple samples (Figure S11).

### Pellets Microstructure

We look at the materials microstructure to understand the underlying reasons governing the electronic and phononic transport. Scanning electron microscopy (SEM) analysis of fractured pellet surfaces reveals distinct differences in grain sizes among the samples. Specifically, all Ag_2_Se-CdSe NCPs exhibit smaller grain sizes compared to the untreated Ag_2_Se, and the average grain size decreases with increasing CdSe content (Figure S12).

To identify additional differences, we use transmission electron microscopy (TEM) on the untreated Ag_2_Se and the Ag_2_Se-7%CdSe NCP. The untreated Ag_2_Se sample contains randomly distributed Ag nanocrystals (NCs) ranging from 10 to 50 nm in size (Figure 4A) as evidenced by scanning TEM energy dispersive X-ray spectroscopy (STEM-EDX) elemental mapping (Figures 4B–C) and high-resolution TEM (HRTEM) analysis (Figure S13). Such secondary phase Ag NCs have been previously reported.^10,14,28,39^ In contrast, no secondary Ag phases are observed in the Ag_2_Se-7%CdSe NCP (Figures 4G-J). Instead, the microstructure exhibits a higher defect density compared to the untreated Ag_2_Se. In particular, a larger number of dislocations are present (Figures 4H–I, S14). These features contribute to the reduction of the carrier concentration and the larger absolute values of the S in all NCPs. It is known that the presence of dislocations can decrease the doping efficiency of Ag-interstitials by partial entrapment of these Ag interstitial atoms, impeding their ionization into Ag^+^ and free electrons.^14^ This point is strengthened by the higher *n*_*H*_ of the untreated Ag_2_Se in the superionic high temperature phase, where Ag^+^ ions are highly mobile and contribute to the high *n*_*H*_. Moreover, preventing the formation of Ag NCs during sintering, which act as electron donors, also has an effect on reducing the carrier concentration.

**Figure 4:**
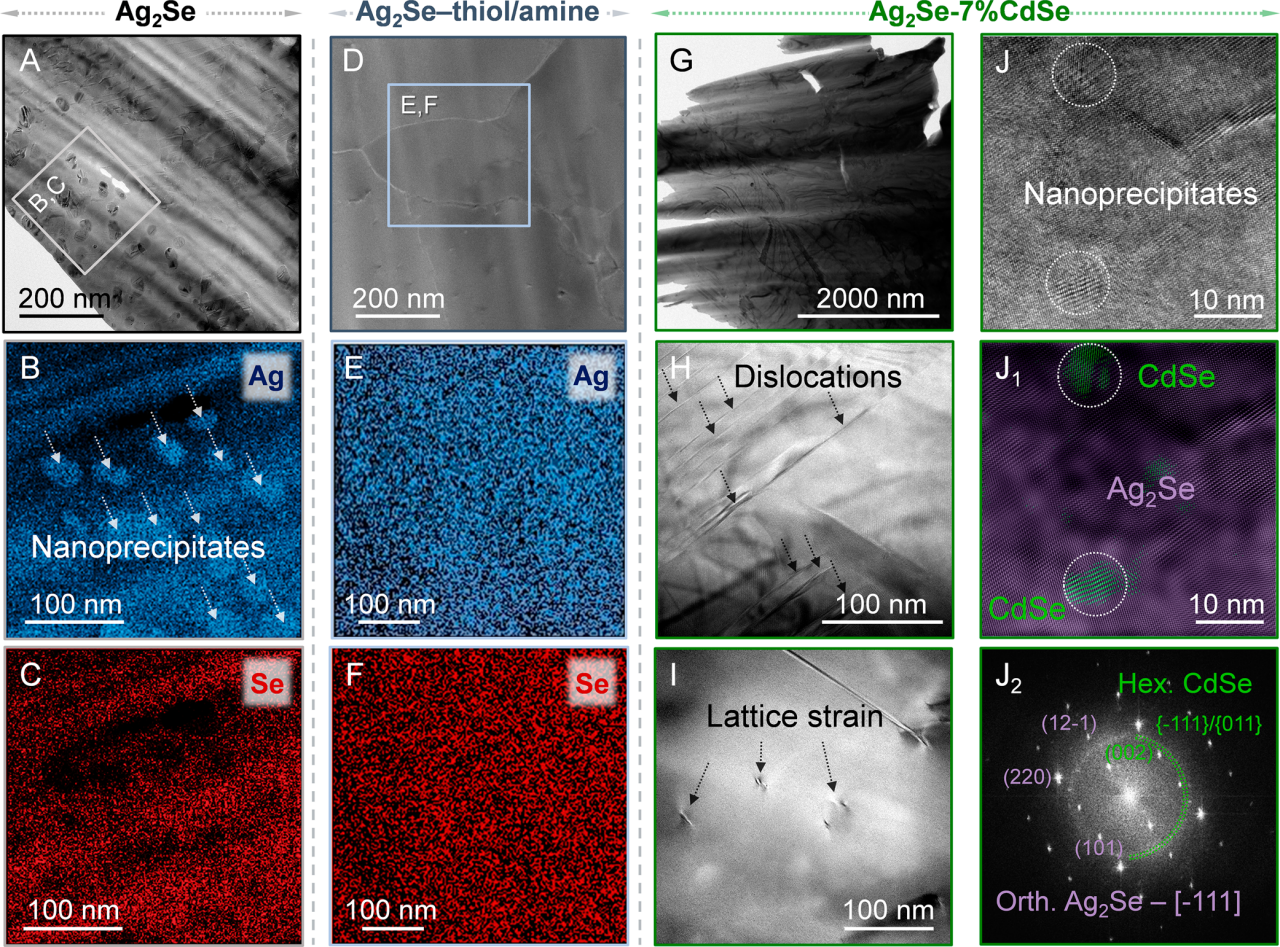
A) Overview TEM image of the untreated Ag_2_Se sample. The TEM image shows the Ag_2_Se matrix with multiple NCs distributed throughout. B–C) STEM-EDX elemental mapping of the region marked by the gray square, with Ag represented as blue and Se as red. D) Overview TEM image of the thiol-amine treated Ag_2_Se sample. The TEM image depicts the Ag_2_Se matrix without NCs distributed in it. E–F) STEM-EDX elemental mapping of the region marked by the light blue square, with Ag shown in blue and Se in red. G) Overview TEM image of the Ag_2_Se-7%CdSe NCPs. H) TEM image showcasing dislocations within the pellet. I) TEM image illustrating lattice strain defects within the material. J) An HRTEM image region showing the presence of small crystallites in the matrix. J_1_) FFT of the entire region, revealing two distinctive features of a spot pattern generated by the matrix grain and a ring pattern generated by the nanocrystallites. J_2_) Inverse FFT highlighting two different phases; the orthorhombic phase of the Ag_2_Se matrix is indicated in purple and the hexagonal phase of CdSe NCs in green.

Moreover, the use of CdSe complexes leads to the formation of CdSe NCs, averaging below 10 nm in size (Figures 4J, S15), dispersed throughout the Ag_2_Se matrix. Their incorporation correlates with a reduction in average grain size (Figure S12). In untreated Ag_2_Se, annealing above the phase transition temperature facilitates grain coarsening via coalescence, driven by the weak bonding nature and high mobility of Ag in the superionic cubic phase. In contrast, the presence of CdSe precipitates limits the Ag mobility and thus mass transport during sintering, as CdSe and Ag_2_Se remain immiscible within the applied temperature range (Figure S7). These microstructural features also impact charge and heat transport. The denser network of high-angle grain boundaries (Figure S12)^58^ and dispersed CdSe NCs (Figures 4J, S15) act as scattering centers for charge carriers, reducing *μ*_*H*_ within the Ag_2_Se-CdSe NCPs. A wide range of phonon scattering sources is present, spanning from atomic-scale defects (e.g., dislocation cores), to dislocation lines and grain boundaries, and up to 3D nanoscale inclusions (CdSe NCs). This hierarchical structure enables scattering of phonons across the full vibrational spectrum, leading to a pronounced suppression of *κ*_*L*_ in the Ag_2_Se-CdSe NCPs compared to the untreated Ag_2_Se (Figure 3E).

### Unveiling the Underlying Chemistry of Surface Treatment

The post-synthetic surface modification of Ag_2_Se with CdSe complexes induced several key differences in the NCP that drive the enhanced thermoelectric performance: (i) the reduction of average grain size, (ii) the disappearance of Ag NCs and (iii) the presence of CdSe NCs.

To unveil the origins of the observed differences in microstructure, composition, and transport properties, we need to determine the reactions taking place at the liquid–solid interface and those occurring during solid-state sintering. The constituents of the reaction medium for the surface treatments are *N*-Methylformamide (MFA), which serves as the solvent, the Ag-rich-surface particles, the CdSe complexes, and the thiol-amine mixture used to form the CdSe complexes.

Since this is a complex system and several reactions can occur simultaneously, we run a series of control experiments. The thiol-amine “alkahest” solvent systems are known for their high dissolution power, capable of dissolving many bulk inorganic materials to yield molecular inks.^59,60^ Therefore, we first examine the liquid reaction medium composed of the residual thiol-amine mixture in MFA and any byproduct of the reaction with the Ag_2_Se particles. Electrospray ionization high-resolution mass spectrometry (ESI-HRMS) reveals the presence of Ag-thiolates, indicating interfacial reactivity. Among those species, we identified a pattern, according to which SC_2_H_4_ units are added within the generalized complexes, that are [AgS_*x*_C_2(x‑1)_H_4(x‑1)_]^−^, where x = 2–7, [Ag_2_S_*x*_C_2(x‑2)_H_4x‑7_]^−^, where x = 6–9, and [Ag_4_S_*x*_C_2(x‑3)_H_4x‑11_]^−^, where x = 5–9 (Table S5). Based on these fragments and the annotation of the complexes [AgS_2_C_2_H_4_]^−^, [Ag_3_S_4_C_4_H_8_]^−^ and [Ag_5_S_6_C_6_H_12_]^−^, we conclude that the parent ion is of larger n and can be generalized as [Ag_2n‑1_S_2n_(C_2_H_4_)_n_]^−^. The presence of Ag-thiolates indicates that Ag is being dissolved from the Ag-rich-surface particles, despite the small content of the “alkahest” solvent used (1.1 mL *per* 2 g Ag_2_Se in the Ag_2_Se-7% CdSe NCP preparation), with the general reaction equation shown below:

3 $$\left(2n-1\right){\mathrm{Ag}}_{\mathrm{surface}}^{+}+nSC_{2}H_{4}S^{2-}\to {\left[{\mathrm{Ag}}_{2n-1}S_{2n}{\left(C_{2}H_{4}\right)}_{n}\right]}^{-}$$

These results are in agreement with the data obtained for the pellets microstructure (Figure 4) and their corresponding transport properties (Figure S16). The temperature dependent σ is reduced compared to the untreated case and is very close to the value of the Ag_2_Se-7%CdSe sample, where the same volume of thiol-amine was used (Figure S16A). All this data clearly reveal that the thiol-amine mixture removes the excess of Ag, yielding a material within the Ag_2_Se solid solution range (Figure 4D-F).

Next, we proceed to understand the role of the CdSe complexes within the final material, its transport properties, and the reactions that yield such results. To the best of our knowledge, there are no reports describing the chemical nature of the CdSe complexes in thiol-amine solvent. Polyanionic chalcogenidometallates of various chain lengths and rich in chalcogen have been reported in other solvent systems.^49,59,61,62^ We performed ESI-HRMS on isolated complexes, redispersed in Dimethyl sulfoxide (DMSO), to characterize the species formed in our system (Table S6). The spectra reveal a series of selenium-rich selenidocadmate complexes, including [Cd­(Se_4_)_2_]^2–^_,_ along with fragments such as [CdSe_7_]^−^, [CdSe_6_]^−^ and [CdSe_5_]^−^, and [CdSe_4_]^−^ suggesting that [Cd­(Se_4_)_2_]^2–^ is the dominant or parent species. We also observe free Se_3_^–^ anions and minor peaks assignable to mixed Se/S species such as [Cd­(Se_3_S)_2_]^2–^. Based on prior studies of Se chemistry in similar solvents,^63,64^ the higher solubility of Se compared to Cd in the solvent mixture,^65^ and the detection of polyselenides by ESI-HRMS, we propose that the reaction starts with the formation of polyselenide anions (eq 4). These species then react with CdO and the thiol/thiolate ligand to form polyanionic CdSe complexes (eq 5), without any solid byproduct.

4 $$\mathrm{nSe}+\mathrm{thiolates}\to {\mathrm{Se}}_{x}^{-}+\mathrm{disulfide}/\mathrm{thiolate}/\mathrm{Se adducts}$$

5 $$\mathrm{CdO}+{\mathrm{Se}}_{x}^{-}+\mathrm{thiol}/\mathrm{thiolate}\to {\left[\mathrm{Cd}{\left({\mathrm{Se}}_{4}\right)}_{2}\right]}^{2-}+H_{2}O+\mathrm{thiol}/\mathrm{thiolate}/\mathrm{Se byproducts}$$

We then propose that these [Cd­(Se_4_)_2_]^2–^ complexes act as nucleophiles, attacking excess Ag^+^ at the particles surface to form solid Ag_2_Se (eq 6).

6 $${\left[\mathrm{Cd}{\left({\mathrm{Se}}_{4}\right)}_{2}\right]}^{2-}+2{\mathrm{Ag}}_{\mathrm{surface}}^{+}\to {\mathrm{Ag}}_{2}\mathrm{Se}\;\left(\mathrm{solid}\right)+\mathrm{CdSe}\;\left(\mathrm{solid}\right)+\mathrm{Se byproducts}\;\left(\mathrm{solution}\right)$$

Although the precise speciation of Se in the liquid phase is not fully resolved, this does not limit our interpretation of the reaction outcome. The data clearly demonstrate that CdSe complexes in solution react with surface Ag^+^, resulting in the formation of CdSe and additional Ag_2_Se. This process effectively removes excess Ag from the particles and tunes the final composition of the solid.

To demonstrate the feasibility of reaction eq 6, we treat Ag_2_O powders with isolated CdSe complexes in DMSO. This approach excludes the presence of free thiol-amine and avoids the masking of newly formed Ag_2_Se by pre-existing Ag_2_Se. The reaction time and powder purification steps are kept the same. Upon treatment with the CdSe complexes, the powder changes color from gray (Ag_2_O) to black indicating the formation of Ag_2_Se, which was further verified by XRD (Figure S17A). The XRD pattern also reveals the presence of crystalline CdSe, which is confirmed by TEM analysis of the resulting powder (Figure S18). The crystallization of cadmium metallates has been previously reported, usually upon heating,^49,65^ but here in the presence of Ag_2_O or Ag_2_Se they crystallize at room temperature. Chalcogenidometallates typically require an excess of chalcogen to ensure stability as they create a nucleophilic coordination environment.^66^ However, when new Ag_2_Se is formed, the chalcogen content is depleted destabilizing the CdSe complexes and yielding crystalline CdSe and polyselenides (Figure 5). Concerning the mixed Cd–Se–S complexes, we have not identified any Ag_2_S formation and believe it is unlikely due to its reported stability as dissolved complexes in the “alkahest” solvent system.^67^

**Figure 5:**
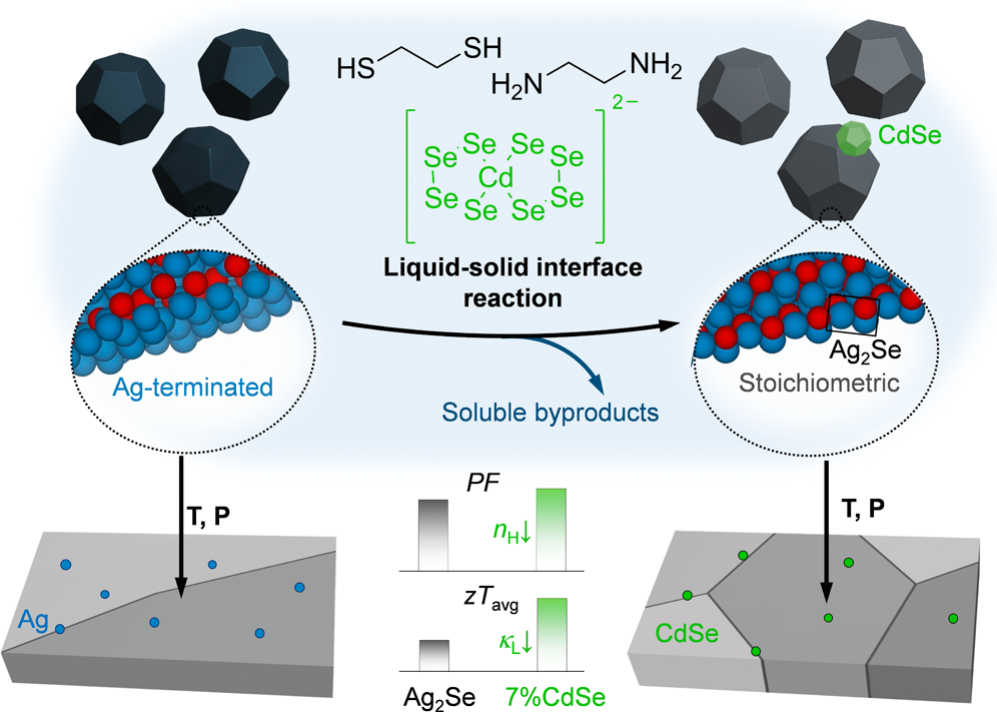
Proposed reaction scheme derived from control experiments, structural characterization and transport properties.

Finally, an additional possible reaction is between the CdSe complexes, and the *in situ* formed Ag-thiolates. As a control experiment, isolated Ag-thiolates prepared from Ag_2_O were mixed with CdSe complexes, resulting in immediate precipitation. XRD analysis confirmed the powder to be Ag_2_Se, showing no reflections from CdSe (Figure S17B). The liquid phase contains Cd-thiolates [Cd­(S_*x*_C_2(x‑2)_H_4(x‑2)+1_)]^−^, with x = 3–13 (Table S7), and unreacted Ag-thiolates, indicating that Cd-thiolates are formed during the reaction (eq 7). The structural similarity between the Cd-thiolates [e.g., Cd­(S_6_C_8_H_17_)]^−^ and Ag-thiolates [e.g., Ag_2_(S_6_C_8_H_17_)]^−^ supports the plausibility of this pathway (Table S5). A possible reaction is depicted below:

7 $${\left[\mathrm{Cd}{\left({\mathrm{Se}}_{4}\right)}_{2}\right]}^{2-}+\mathrm{Ag}-\mathrm{thiolates}\to {\mathrm{Ag}}_{2}\mathrm{Se}\left(\mathrm{solid}\right)+\mathrm{Cd}-\mathrm{thiolates}\;\left(\mathrm{solution}\right)+\mathrm{byproducts}\;\left(\mathrm{solution}\right)$$

In the Ag_2_Se-CdSe NCPs, neither Ag_2_S, nor CdS phases have been detected in the structural or elemental analysis, suggesting that both Cd-thiolates and Ag-thiolates remain in solution phase and are removed during powder purification.

Overall, the surface modification of Ag_2_Se particles with CdSe complexes containing residual “alkahest” solvent in MFA triggers a series of reactions that produce newly formed Ag_2_Se, with the Ag source originating from the excess Ag present on the surface of the as-synthesized particles, while CdSe also forms as part of the reaction sequence. Both of these facts significantly affect the final material, explaining the previously described differences in microstructure, composition and transport properties of the NCPs versus the untreated Ag_2_Se or the Ag_2_Se treated with only the “alkahest” solvent. Specifically, the removal of excess Ag, resulting in stoichiometric Ag_2_Se, leads to a reduced *n*_*H*_, closer to the optimal value for Ag_2_Se. Simultaneously, the presence of CdSe NCs contributes to a decrease in average grain size, which scales with CdSe content. This effect arises from the immiscibility of CdSe and Ag_2_Se within the relevant temperature range (Figure S7), which inhibits Ag^+^ diffusivity in the high temperature cubic phase.

## Conclusions

In summary, we have demonstrated that liquid–solid interface reactions between Ag_2_Se particles and CdSe complexes in a thiol-amine medium enable precise control over stoichiometry, defect populations, and grain size; factors that are otherwise difficult to regulate in Ag_2_Se due to its narrow single-phase range and high Ag^+^ diffusivity. We show that thiol-amine solvents react with surface Ag^+^ to form soluble Ag-thiolate species, while polyanionic CdSe complexes react with surface Ag^+^ to nucleate both new Ag_2_Se and CdSe NCs. The changes in the powder properties due to the liquid–solid interface reaction results in a material constituted of a single-phase Ag_2_Se matrix with a high density of grain boundaries and dislocations decorated with CdSe NCs.

The removal of excess Ag prevents phase segregation during sintering and reducing *n*_*H*_, while CdSe NCs inhibit grain coalescence, reducing *κ*_*L*_ through phonon scattering by structural features ranging from atomic to nanoscale dimensions (dislocations, grain boundaries, CdSe NCs). As a result, the Ag_2_Se-CdSe nanocomposite prepared via this low-temperature, solution-phase protocol achieves a reproducible *zT* of ≈1.04 from 300 to 390 K, comparable to state-of-the-art n-type materials, but obtained using inexpensive precursors, simple solvents, and mild processing. Beyond these performance gains, our work provides a clear mechanistic framework for rational, design-driven interfacial engineering, suggesting that similar strategies could be applied to optimize other material systems.

## Supplementary Material



## Notes

Details of additional materials characterization (X-ray diffraction patterns, SEM, TEM, extra electrical and thermal characterization), stability measurement, repeatability, and comparison with literature values. (PDF)

## ⊥

Department of Physics and Astronomy “Ettore Majorana”; and IMM-CNR, University of Catania, S. Sofia 64, 95123 Catania, Italy
