# Supplementary material for: Liquid–Solid Interface Reactions Drive Enhanced Thermoelectric Performance in Ag2Se
Source: J Am Chem Soc. 2025 Aug 22;147(35):32199–208. doi: 10.1021/jacs.5c11435 (PMC12412162; doi:10.1021/jacs.5c11435)
Supplement: Supplementary file 1 [file ja5c11435_si_001.pdf]

SUPPORTING INFORMATION

Liquid-Solid Interface Reactions Drive Enhanced Thermoelectric Performance in Ag_2Se

Yu Liu,[†,§,#,*] Tobias Kleinhanns,[§,#] Sharona Horta,[§] Ewelina P. Dutkiewicz-Kopczynska,[§] Shaoqing Lu,[†] Maria Chiara Spadaro,[¶,ǁ] Aziz Genç,[¶] Lei Chen,[♠] Khak Ho Lim,[◊] Min Hong,[♠] Jordi Arbiol,[¶,ʲ] Maria Ibáñez[§,*]

[†] Anhui Province Engineering Research Center of Flexible and Intelligent Materials, School of Chemistry and Chemical Engineering, Hefei University of Technology, 230009, Hefei, China.

[§] Institute of Science and Technology Austria (ISTA), Am Campus 1, 3400, Klosterneuburg, Austria.

[¶] Catalan Institute of Nanoscience and Nanotechnology (ICN2), CSIC and BIST, Campus UAB, Bellaterra, 08193, Barcelona, Catalonia, Spain.

[ǁ] Department of Physics and Astronomy "Ettore Majorana" and IMM-CNR, University of Catania, S. Sofia 64, 95123 Catania, Italy.

[♠] Centre for Future Materials and School of Engineering, University of Southern Queensland, Springfield Central Queensland, 4300, Australia.

[◊] Institute of Zhejiang University-Quzhou, 78 Jiuhua Boulevard North, Quzhou 324000, Zhejiang, China.

[ʲ] ICREA, Pg. Lluís Companys 23, Barcelona, Catalonia, 08010, Spain.

[#] Y.L. and T.K. contributed equally to the work.

* E-mail: Y. Liu: *yliu@hfut.edu.cn,* M. Ibáñez: *mibanez@ist.ac.at*

Content

Methods S3

SEM and EDX characterization of as-synthesized Ag_2Se particles S7

XPS of untreated Ag_2Se particles and CdSe surface modified Ag_2Se particles S8

SEM of untreated Ag_2Se particles and surface modified Ag_2Se particles S8

XRD pattern of CdSe crystallized from molecular complexes S9

XRD pattern of annealed Ag_2Se-x%CdSe powder S10

Rietveld refinement profiles of the Ag_2Se-x%CdSe pellets XRD patterns S11

T-dependent XRD pattern of Ag_2Se and Ag_2Se-7%CdSe NCPs S12

Material stability S15

Repeatability S16

SEM images of the fractured pellets surfaces S17

Ag nanodomains within the untreated Ag_2Se pellet S18

Dense dislocations within the Ag_2Se-7%CdSe pellets microstructure S18

CdSe nanodomains within the Ag_2Se-7%CdSe pellet S19

Transport data for different Ag:Se ratio and with/without en/edtH_2 treatment S20

XRD pattern of Ag_2O powder and Ag-thiolates treated with CdSe complexes S21

In-situ TEM heating experiment showing CdSe redistribution with annealing S22

Pellet density and composition S23

Lattice parameters S24

Room-temperature hole concentration (n_H) and mobility (μ_H) S24

Ag-thiolate complexes S25

CdSe complexes S26

Cd-thiolate complexes S27

Theoretical calculation details S28

Weight mobility and Phonon density of states (PDOS) calculations S29

Quality factor calculation S29

References S30

Methods

***Chemicals**:* Silver (I) nitrate ($AgNO_3$, AR), sodium borohydride ($NaBH_4$, 98%), cadmium (II) oxide (CdO, 99.99%), and N-methylformamide (MFA, 99%) were purchased from Fisher Scientific. Selenium powder (Se, 100 mesh, ≥99.5%), ethylenediamine (en, 99%), 1, 2-ethanedithiol ($edtH_2$, ≥95.0%), anhydrous dimethyl sulfoxide (DMSO, ≥99.9%), anhydrous acetone (extra dry) and ethanol (95%) were purchased from Sigma-Aldrich. All chemicals were used as received without further purification.

Large-scale synthesis of surfactant-free Ag_2Se particles: The Ag_2Se particles were synthesized according to a procedure previously reported by Han *et al.*,[1] with slight modifications. A typical experimental procedure was as follows; preparation of the Ag precursor stock solution A: 60 mmol of $AgNO_3$ (10.1922 g) were dissolved in 150 mL of deionized water in a 500 mL three-neck round-bottom flask at room temperature and under Ar flow, forming a transparent solution. In parallel, the Se precursor stock solution B was prepared by first dissolving 60 mmol of $NaBH_4$ (2.2698 g) in 150 mL of deionized water in a 250 mL three-neck flask. Then, 33 mmol of Se powder (2.6057 g) was slowly added without stirring, as the formation of NaHSe released hydrogen gas. After around 5-10 min, the mixture was stirred again under Ar flow, forming a transparent solution. Then, solution A was heated to its boiling point (100°C), while assuring reflux using a condenser. Once this temperature was reached, the freshly prepared solution B was quickly injected into solution A, which immediately changed color, from transparent to black. Upon injection, the temperature of the reaction mixture dropped to *ca.* 80°C. As soon as the reaction mixture reaches 100°C again, it was and maintained at this temperature for 30 min. Thereafter, the magnetic stirring was stopped, and the transparent supernatant solution was discarded. The remaining around 60 mL of crude solution was transferred equally into three centrifuge tubes. The particles were then purified for four times using deionized water (35 mL) and ethanol (40 mL) alternately. First, the fresh deionized water was added to the crude solution, and the particles were separated from the liquid phase by centrifugation at 5000 rpm for 1 min. In the second step, ethanol was used to re-disperse the particles, and the solution was centrifuged at 6000 rpm for another 1 min. For the remaining steps, the particles were purified following the same procedure as in steps one and two. The wet particles were then vacuum dried overnight at room temperature. The dried

particles were stored in a nitrogen-filled glovebox until further use. This synthesis protocol was optimized to yield over 8.0 g of Ag_2Se particles per batch, providing enough material for a complete characterization at the laboratory scale.

Molecular complexes preparation: The CdSe molecular complexes were prepared using a modified methodology, as initially developed by R. L. Brutchey *et al.*[2] In this procedure, CdSe molecular complexes at a concentration of 87 mg/mL were prepared by dissolving stoichiometric amounts of CdO (4 mmol) and Se powder (4 mmol) in a mixture of en (8 mL) and edtH_2 (0.8 mL) in a N_2-purged vial. The mixture was agitated at room temperature for *ca.* 5 min until complete dissolution. Owing to their limited stability, all solutions of CdSe molecular complexes were prepared freshly prior to their reaction with Ag_2Se particles in MFA.

Particle surface modification: To fabricate Ag_2Se-x%CdSe (x=2, 4, 6, 7 and 10 in molar percent) nanocomposites (NCPs), varying volumes of the CdSe molecular complexes solution (2%: 0.30 mL, 4%: 0.60 mL, 6%: 0.90 mL, 7%: 1.05 mL, and 10%: 1.50 mL) were initially blended with approximately 50 μL/mL of MFA in N_2-purged vials. Subsequently, these solutions were mixed with 2.0 g of dried Ag_2Se particles in N_2-filled vials. The dispersions were then vigorously stirred at room temperature for 24 h to ensure complete reaction between the CdSe molecular complexes and the Ag_2Se particles. Following the reaction, acetone was added to the mixtures to facilitate the precipitation of the surface modified particles. Inside the nitrogen-filled glovebox, the particles were separated from the liquid phase by centrifugation. These CdSe molecular complexes-treated Ag_2Se particles, were re-dispersed in acetone followed by centrifugation, and were then vacuum-dried to yield a fine powder, which was subsequently stored inside the glovebox until future use.

Bulk nanomaterial consolidation: Dried Ag_2Se-x%CdSe (x=0, 2, 4, 6, 7 and 10) NCPs were first annealed at 400 °C for 60 min under a N_2 gas flow inside a tube furnace with the heating rate of *ca.* 10 °C/min. Subsequently, the annealed powders were ground into fine powder using an agate mortar inside a nitrogen-filled glovebox to prevent oxidation. The resulting powders were transferred into a graphite die and sintered under vacuum in an AGUS PECS SPS System (Model SPS 210Sx), where a pressure of 45 MPa and a temperature of 400 °C were applied for 5 min to produce pellets with a diameter of 8.6 mm and a thickness of 1.5 mm. The relative densities of

the sintered pellets were measured using the Archimedes' method, indicating that all samples obtained densities exceeding 95% of their theoretical values.

Structural and chemical characterization: X-ray diffraction (XRD, 2θ range: 20° to 80°; scanning rate: 5°/min) was conducted using a Bruker AXS D8 ADVANCE X-ray diffractometer with Cu–Kα radiation (λ = 1.5406 Å). Temperature-dependent XRD was also performed on sintered Ag_2Se and Ag_2Se-7%CdSe pellets using the Bruker AXS D8 ADVANCE system with height corrections. Temperatures were ramped at 50°C/min, operating at 40 kV, from 323 K to 503 K, and then held isothermally for 20 min at each temperature. Following equilibration, the sample height was aligned, and diffraction data were collected from 20° to 60° in 2θ at a scanning rate of 5°/min. The size and morphology of the initial particles and sintered pellets were examined using field-emission scanning electron microscopy (SEM) on an Auriga Zeiss, operated at 5.0 kV. High-resolution transmission electron microscopy (HRTEM) characterization of Ag_2Se and Ag_2Se-7%CdSe sample was performed using a TECNAI F20 microscope, operated at 200 kV, with a point-to-point resolution of 0.14 nm. The material composition was investigated by inductively coupled plasma optical emission spectroscopy (ICP-OES) on the ICPE-9820 system. Electrospray ionization high resolution mass spectroscopy (ESI-HRMS) was performed on a QExactive HF mass spectrometer (Thermo Fisher Scientific), equipped with a heated electrospray ionization (HESI) source. Source parameters were set to: spray voltage 2.5 kV, capillary temperature 250 °C, sheath gas flow rate 5 a.u., aux gas flow rate 3 a.u., S-lens RF level 50 V. Samples were dissolved in DMSO, and diluted with ACN (DMSO: ACN 1:1), to a concentration of 0.1 mg/mL. In order to avoid possible decomposition products, the sample dilution was performed in a N_2 filled glove box, and the sample was directly infused into the MS instrument at 50 μL/min flow rate. Data were acquired in negative-ion mode at a resolution of 240,000 or 60,000 with AGC target set to $1e^6$ in the measurement range from 100-1000 *m/z* (or narrowed if needed). The final spectra were created as the average from 20 scans, and they were annotated using the FreeStyle software, version 1.8.

Thermoelectric (TE) property measurement: The Seebeck coefficients (*S*) were measured using a static DC method, while electrical resistivity was determined through the standard four-probe approach. Both parameters were measured simultaneously using an LSR-3 LINSEIS system over

a temperature range from ambient to 443 K in a controlled helium (He) atmosphere. Given the precision of the system and the meticulous measurement of dimensions, the estimated error margin for both electrical conductivity (σ) and the Seebeck coefficient (S) was approximately 4%. Consequently, the uncertainty associated with the power factor (PF) is estimated to be around 12%. Thermal conductivity (κ_{tot}) was calculated using the formula $\kappa_{tot} = \lambda C_p \rho$, where λ represents thermal diffusivity, C_p denotes specific heat capacity at constant pressure, and ρ signifies the mass density of the specimen. Thermal diffusivity (λ) was measured using an LFA 1000 Laser Flash Apparatus, with an estimated error of about 5%. The specific heat capacity (C_p) was inferred from empirical relations following the Dulong–Petit law, while density (ρ) measurements were accurately conducted using the Archimedes' method with an estimated error of approximately 2%. Therefore, the overall uncertainty for all parameters involved in calculating the dimensionless figure of merit (zT) is estimated to be around 17%. For clarity, error bars were omitted from the plots. Additionally, Hall charge carrier concentrations (n_H) and mobilities (μ_H) were measured using the Van der Pauw method, with a magnetic field strength of 0.6 T (ezHEMS, NanoMagnetics), including room-temperature data for Ag_2Se-x%CdSe (x=0, 2, 4, 6, 7, and 10) and temperature-dependent data from 300 to 433 K for the untreated Ag_2Se and the Ag_2Se-7%CdSe pellets. The reported values represent the average of 10 measurements, with an estimated error of approximately 10%.

SEM and EDX characterization of as-synthesized Ag_2Se particles

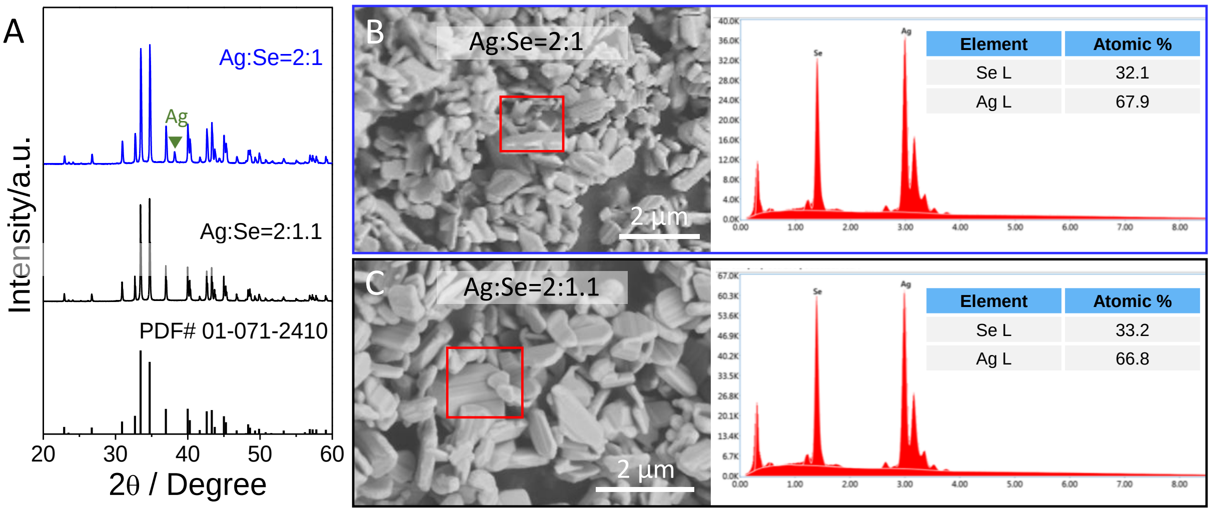

Figure S1. A) XRD patterns of as-synthesized Ag_2Se particles prepared with Ag:Se precursor molar ratios of 2:1 and 2:1.1, respectively. Grey vertical lines correspond to the standard diffraction data for orthorhombic Ag_2Se (PDF# 01-071-2410); Representative SEM images and corresponding elemental compositions determined by SEM-EDX for Ag_2Se particles synthesized with Ag:Se ratios of B) 2:1 and C) 2:1.1, respectively.

XPS of untreated Ag_2Se particles and CdSe surface modified Ag_2Se particles

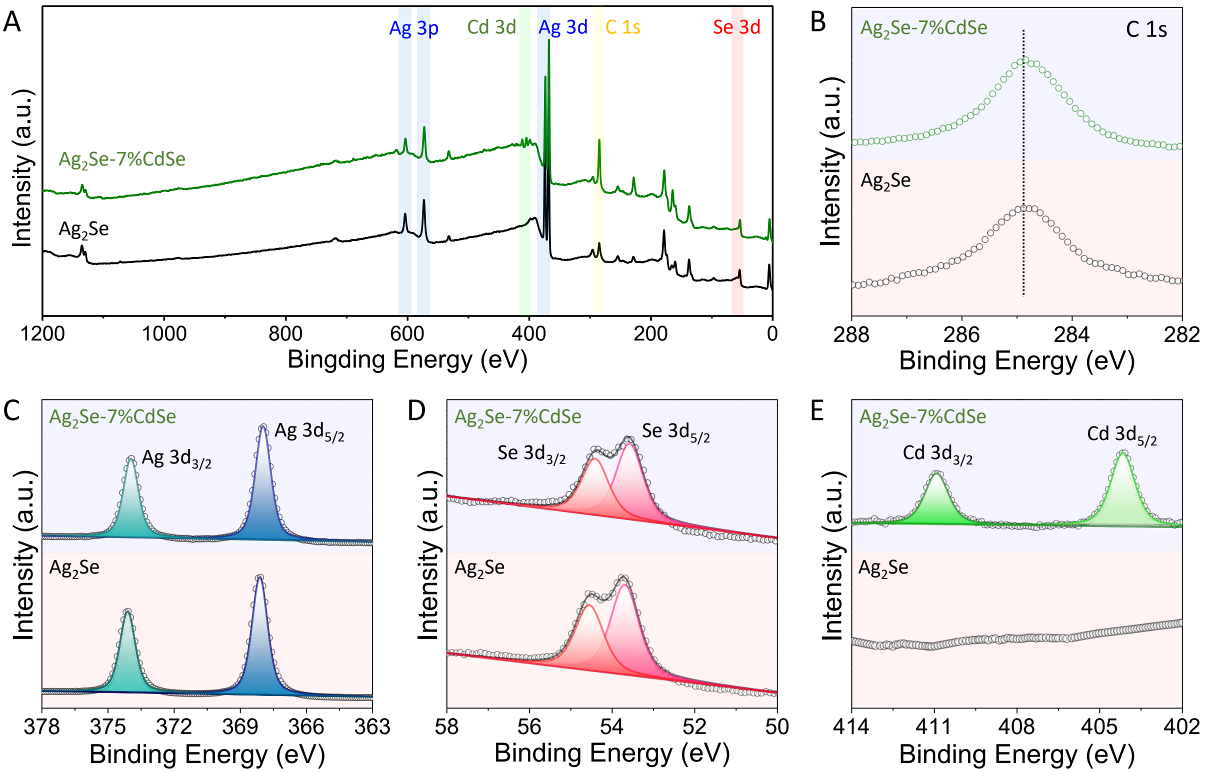

Figure S2. A) XPS survey spectra of as-synthesized Ag_2Se and Ag_2Se-7%CdSe particles after treatment, along with their corresponding high-resolution XPS spectra for B) C 1s, C) Ag 3d, D) Se 3d, and E) Cd 3d.

SEM of untreated Ag_2Se particles and surface modified Ag_2Se particles

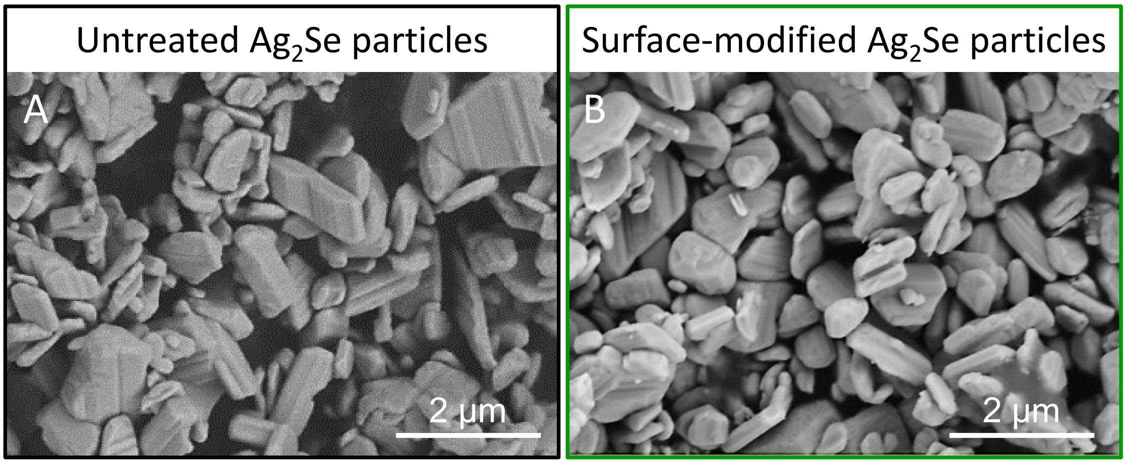

Figure S3. SEM micrographs of A) untreated Ag_2Se particles and B) surface modified Ag_2Se particles.

XRD pattern of CdSe crystallized from molecular complexes

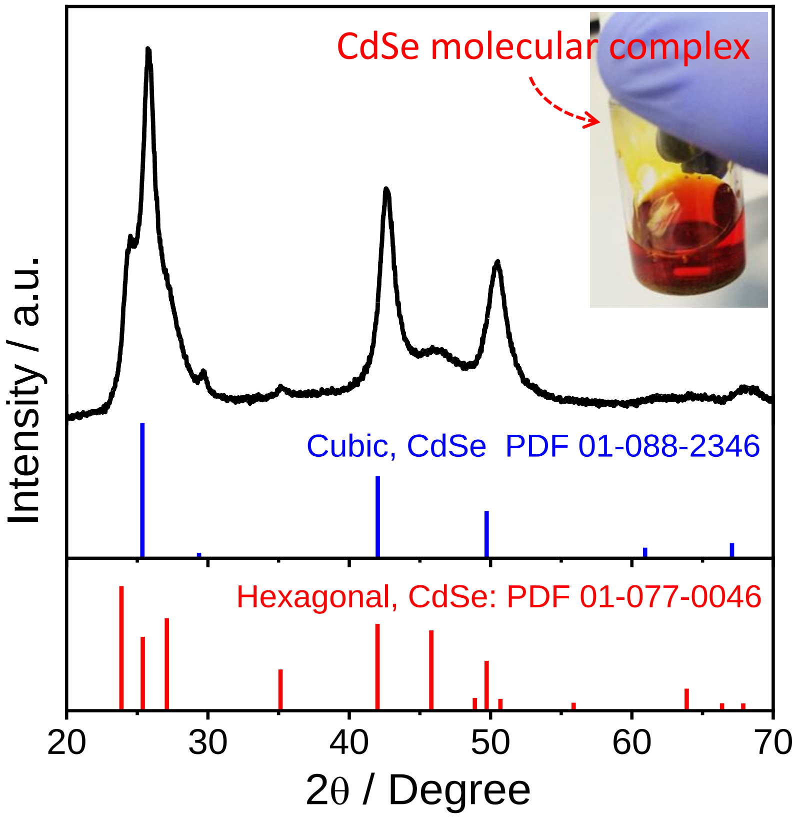

Figure S4. XRD pattern of crystallized CdSe upon annealing the molecular complex at 350°C. The inset shows the initial CdSe molecular complexes solution in the "en-edtH_2" mixture including reference.[3]

XRD pattern of annealed Ag_2Se-x%CdSe powder

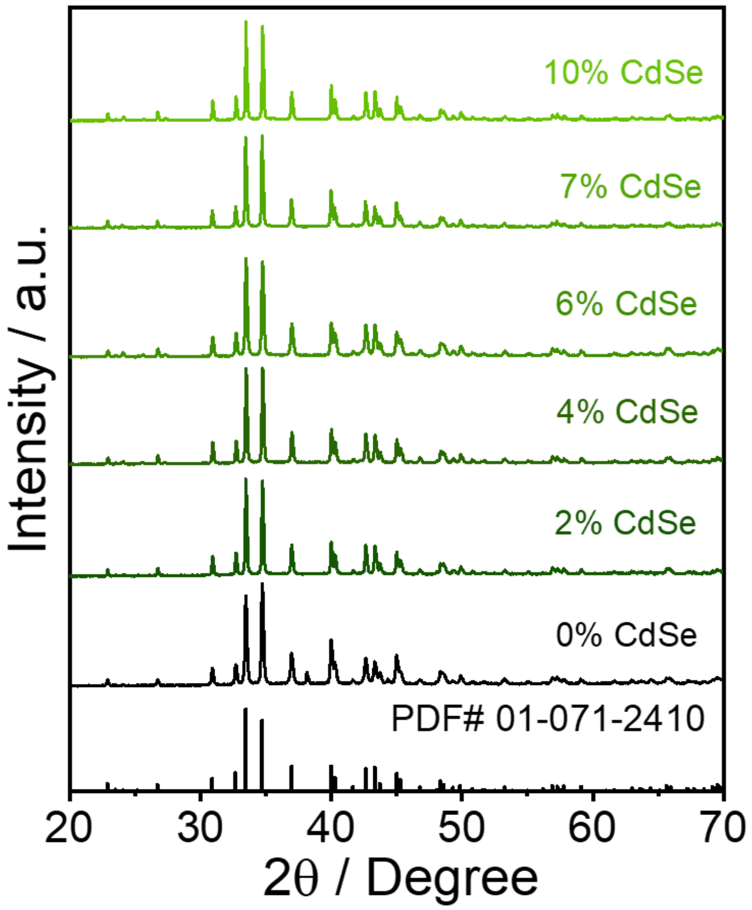

Figure S5. XRD patterns of annealed Ag_2Se-x%CdSe (x=0, 2, 4, 6, 7 and 10) powder. The vertical lines correspond to reference literature data for orthorhombic Ag_2Se (PDF# 01-071-2410).

Rietveld refinement profiles of the Ag_2Se-x%CdSe pellets XRD patterns

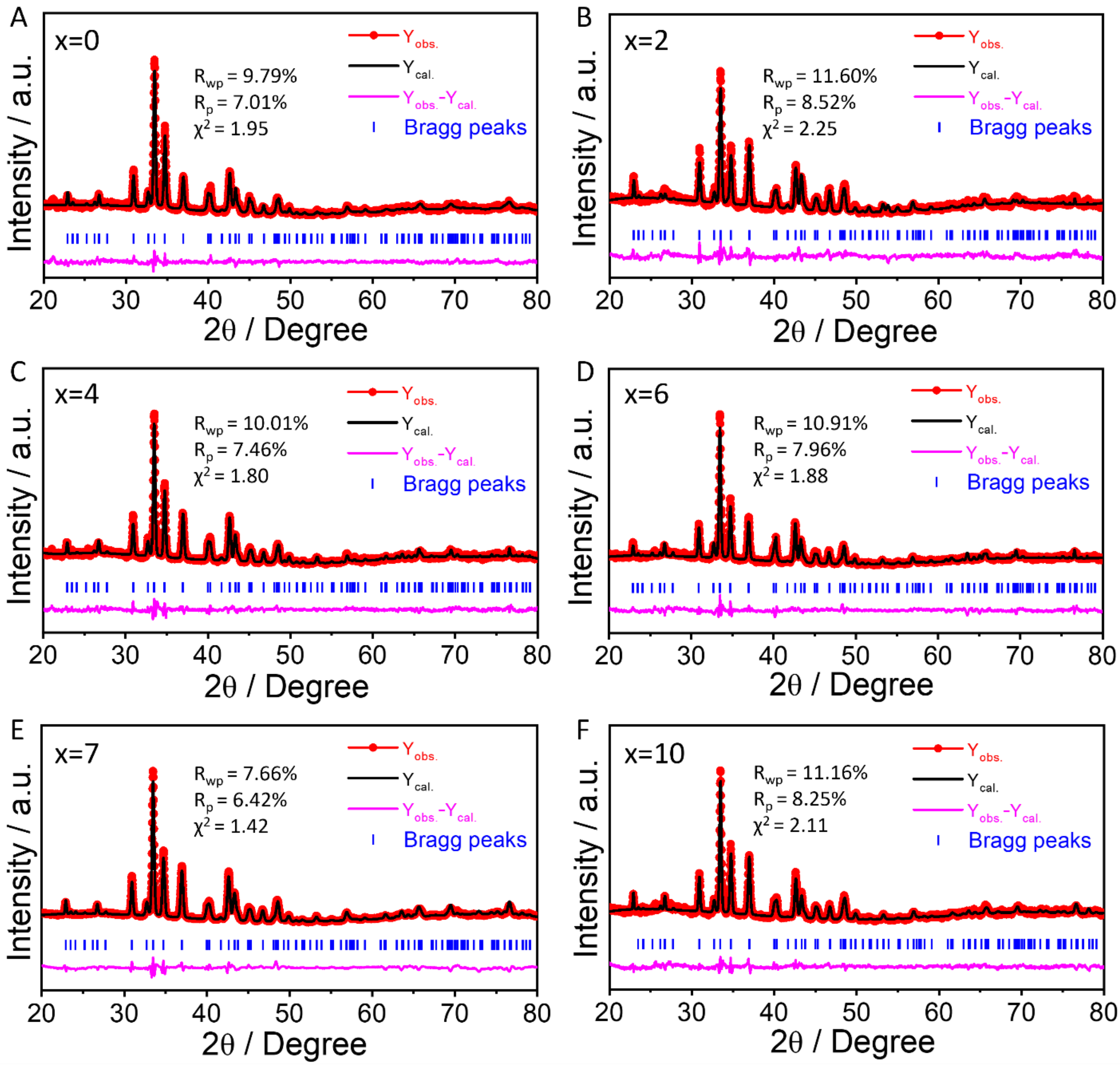

Figure S6. Rietveld refinement profiles of the XRD patterns for Ag_2Se-x%CdSe pellets with x = 0 (A), 2 (B), 4 (C), 6 (D), 7 (E), and 10 (F), performed using GSAS-II software. Red circles represent the observed intensities ($Y_{obs.}$), black lines indicate the calculated patterns ($Y_{cal.}$), and purple lines show the difference curves ($Y_{obs.}$-$Y_{cal.}$). Blue tick marks denote the Bragg reflection positions for orthorhombic Ag_2Se. In the refinement model, the atomic occupancies of Ag and Se were fixed to 1.000, based on the absence of any experimental evidence for Cd incorporation into the Ag_2Se lattice. The B_{iso} values were refined to stabilize the model and are reported here for completeness rather than for physical interpretation. Importantly, the reliability of the refined lattice constants does not depend on the accuracy of B_{iso}, as unit cell parameters are determined

by Bragg peak positions, which are unaffected by these intensity-related parameters. For Ag_2Se–x%CdSe (x = 0, 2, 4, 6, 7, and 10) the B_{iso} values are: Ag: 3.0–3.8 Å^2; Se: 1.5–2.2 Å^2.

T-dependent XRD pattern of Ag_2Se and Ag_2Se-7%CdSe NCPs

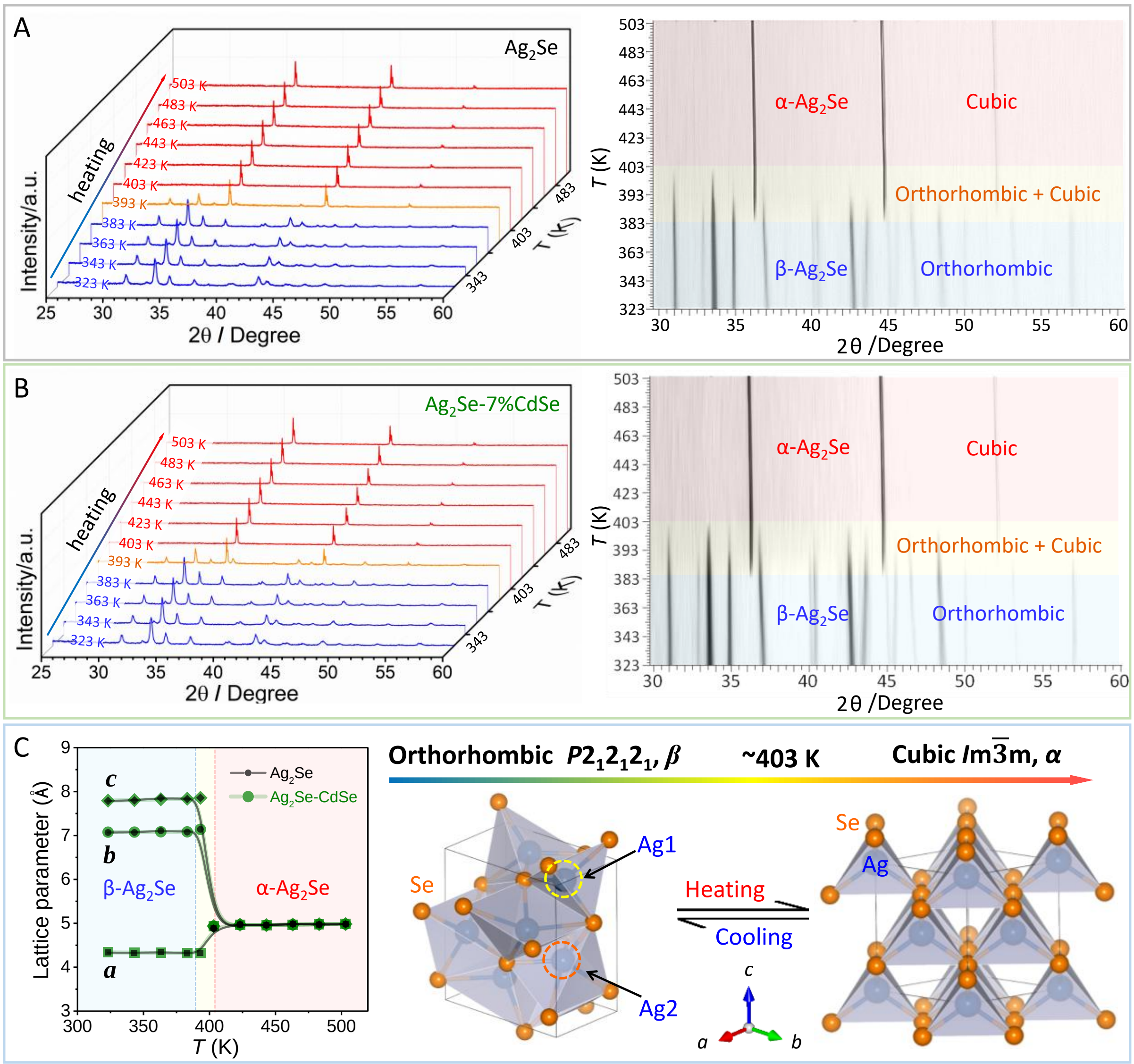

Figure S7. Temperature dependency of the *in situ* XRD patterns and 2D plot of intensity as functions of 2θ and *T* of A) Ag_2Se and B) Ag_2Se-7%CdSe pellets in the temperature range from 323 to 503 K. C) Left: Temperature-dependent lattice parameters as determined by Rietveld refinement of the XRD data. Right: Reversible phase transition of Ag_2Se with different crystal structures between the *β*-Ag_2Se and *α*-Ag_2Se phase.

Additional transport properties

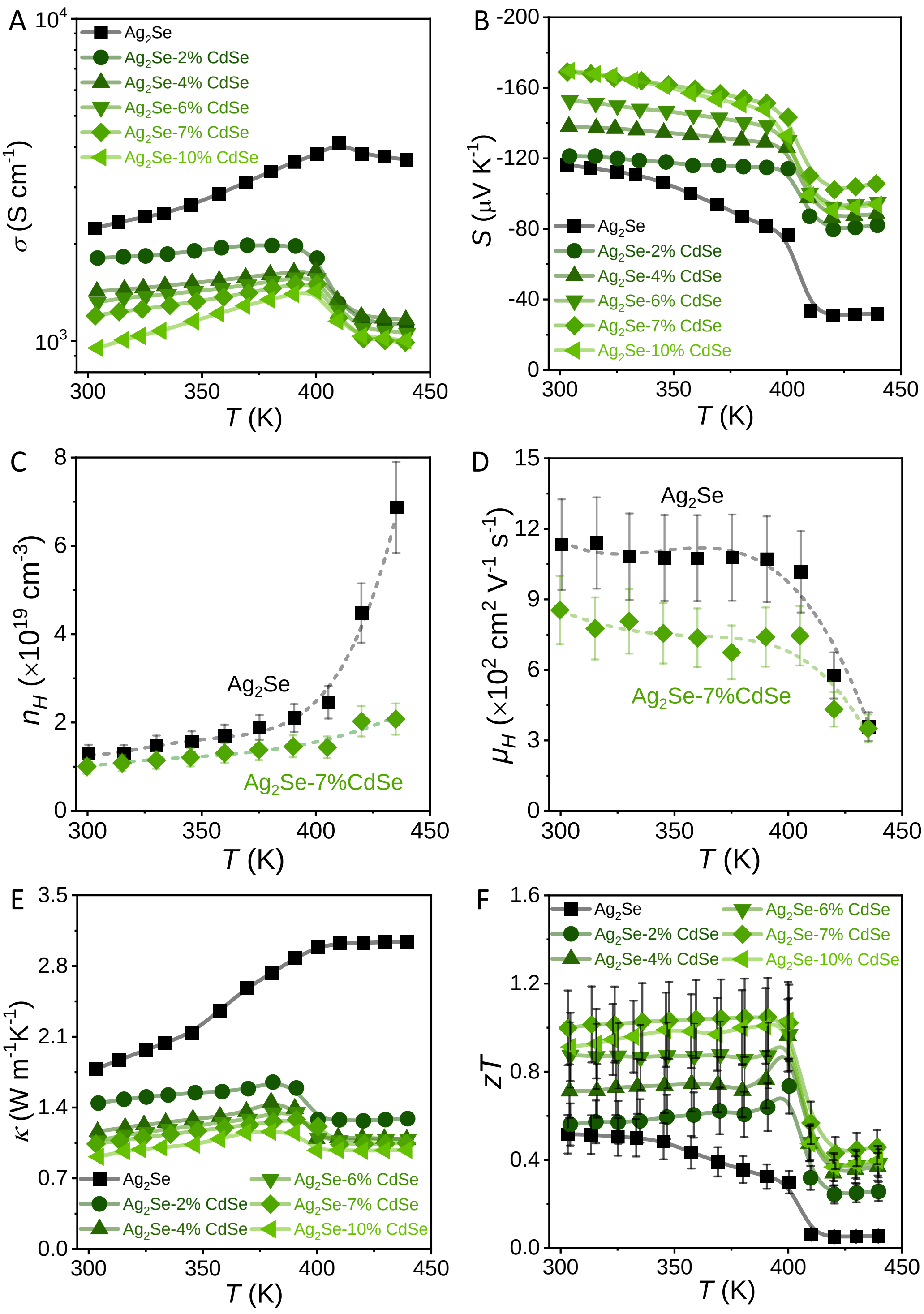

Figure S8. TE properties of Ag_2Se-x%CdSe (x=0, 2, 4, 6, 7 and 10) pellets measured in the temperature range of 300 K-440 K. A) electrical conductivity (σ); B) Seebeck coefficient (S); C-D) full-range temperature-dependent Hall carrier concentration (n_H) and mobility (μ_H) of Ag_2Se and Ag_2Se-7%CdSe; E) thermal conductivity (κ); F) figure-of-merit (zT).

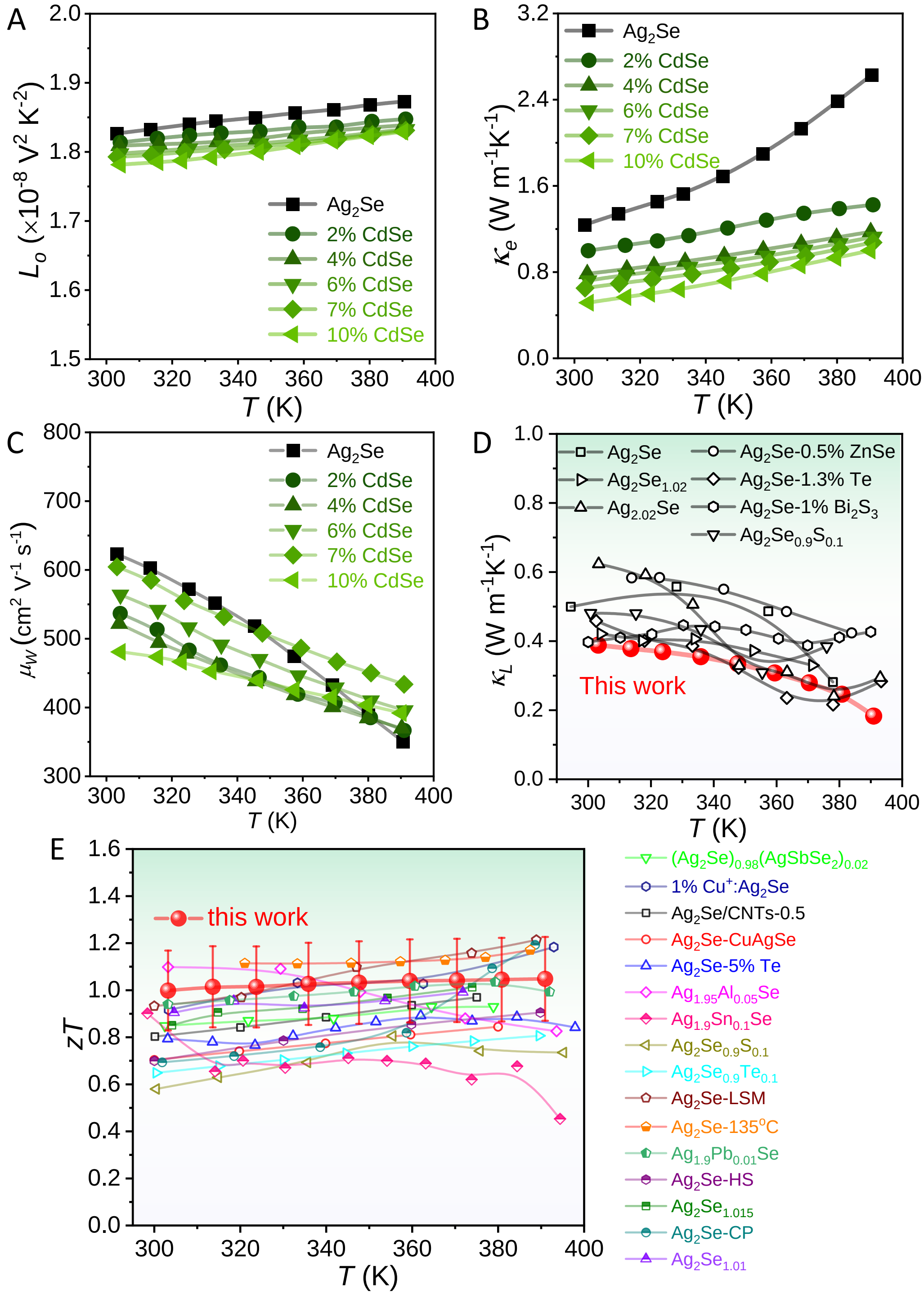

Figure S9. Temperature dependency of A) Lorentz number (L_o), B) electronic thermal conductivity (κ_e), and C) weighted mobility (μ_w) of Ag_2Se-x%CdSe (x=0, 2, 4, 6, 7 and 10) NCPs in the temperature range from 300 K-390 K. D) Comparison of lattice thermal conductivity (κ_L) values of the Ag_2Se-7%CdSe sample with those reported Ag_2Se-based TE materials.[1,4–9] E) Comparison of the temperature-dependent zT values of Ag_2Se-7%CdSe obtained in this study with those of previously reported state-of-the-art Ag_2Se-based TE materials.[4,6,10–23]

Material stability

To verify the thermal stability of the samples, we conducted five consecutive heating and cooling cycles, with temperatures increasing to ~440 K and then decreasing to ~340 K for the Ag_2Se-7%CdSe pellet.

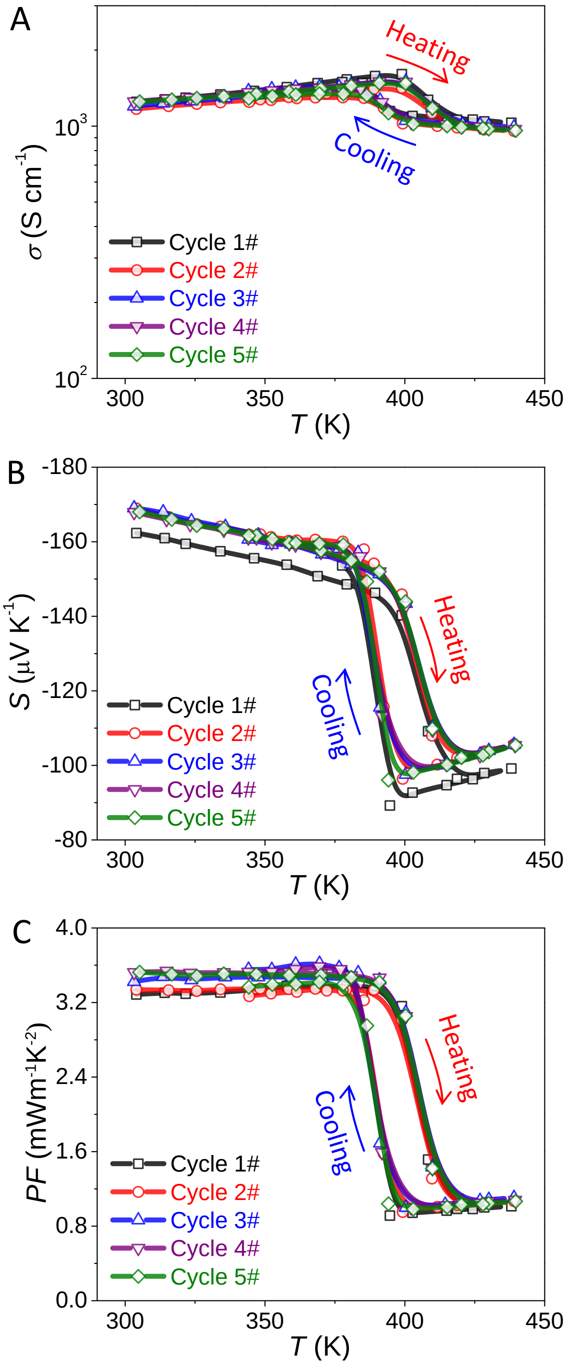

Figure S10. The temperature dependence of A) electrical conductivity (σ); B) Seebeck coefficient (S) and C) power factor (PF) of the Ag_2Se-7%CdSe sample measured five consecutive heating and cooling cycles.

Repeatability

Experiments were conducted to confirm the repeatability of the performance of the Ag_2Se-7%CdSe pellets. Four samples (1#, 2#, 3#, and 4#) from different batches were produced using the same procedure and measured under identical conditions. Slight differences were observed between batches, demonstrating the good repeatability of the samples.

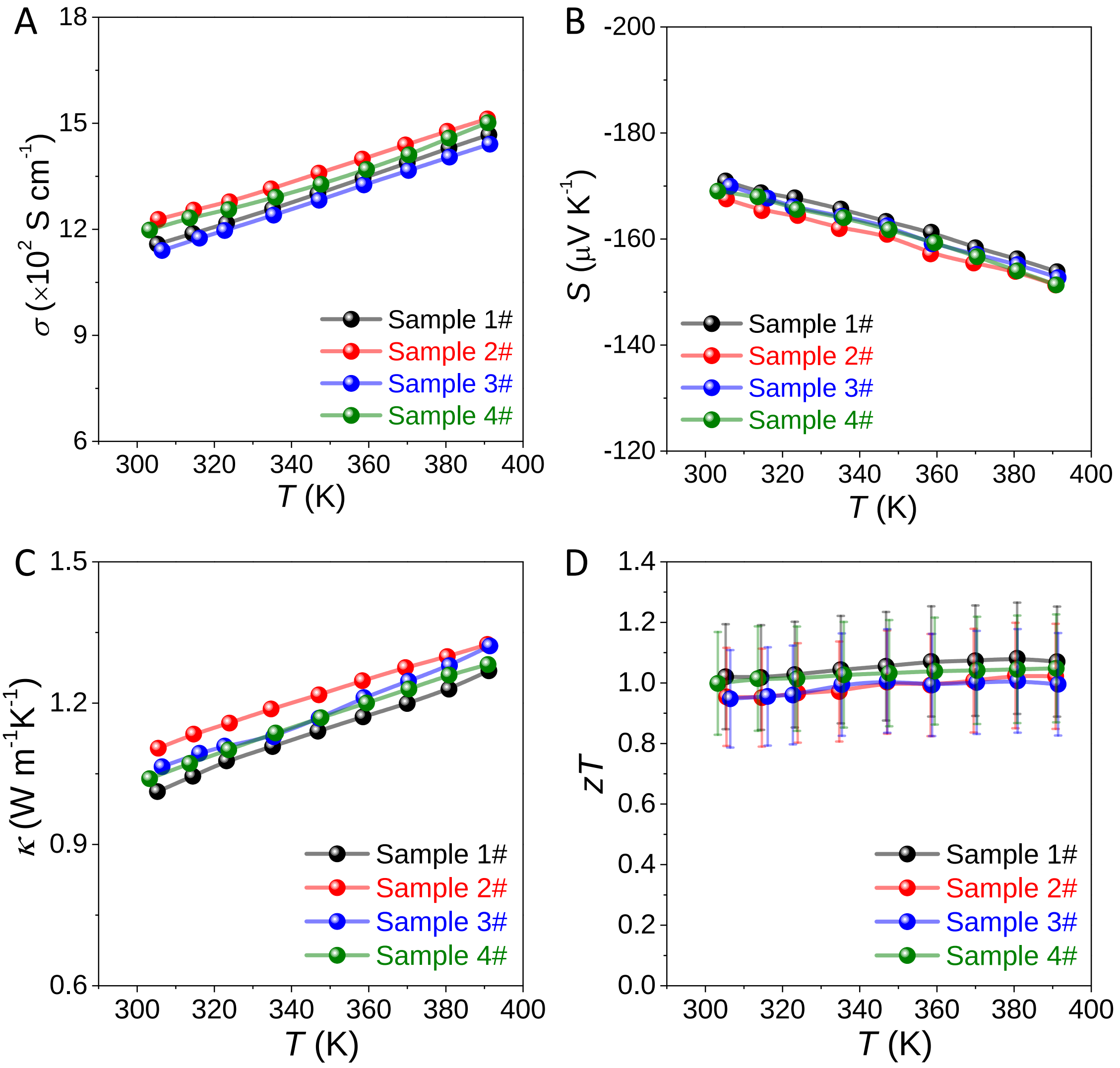

Figure S11. Thermoelectric properties of four Ag_2Se-7%CdSe pellets obtained from four different batches: A) electrical conductivity (σ); B) Seebeck coefficient (S); C) thermal conductivity (κ); and D) figure of merit (zT).

SEM images of the fractured pellets surfaces

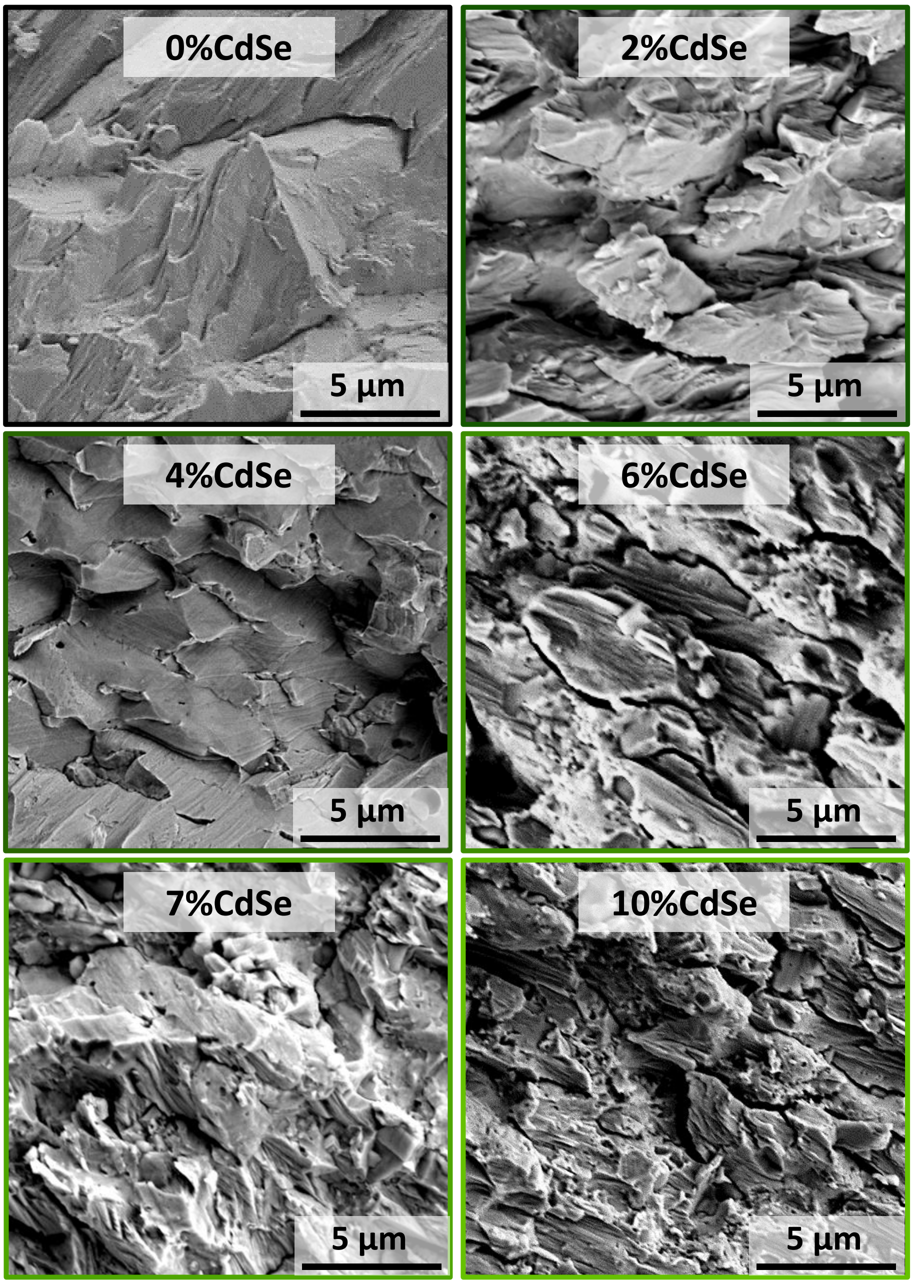

Figure S12. SEM images of the fractured surfaces of sintered pellets prepared from Ag_2Se-x%CdSe (x = 0, 2, 4, 6, 7 and 10) NCPs.

Ag nanodomains within the untreated Ag2Se pellet

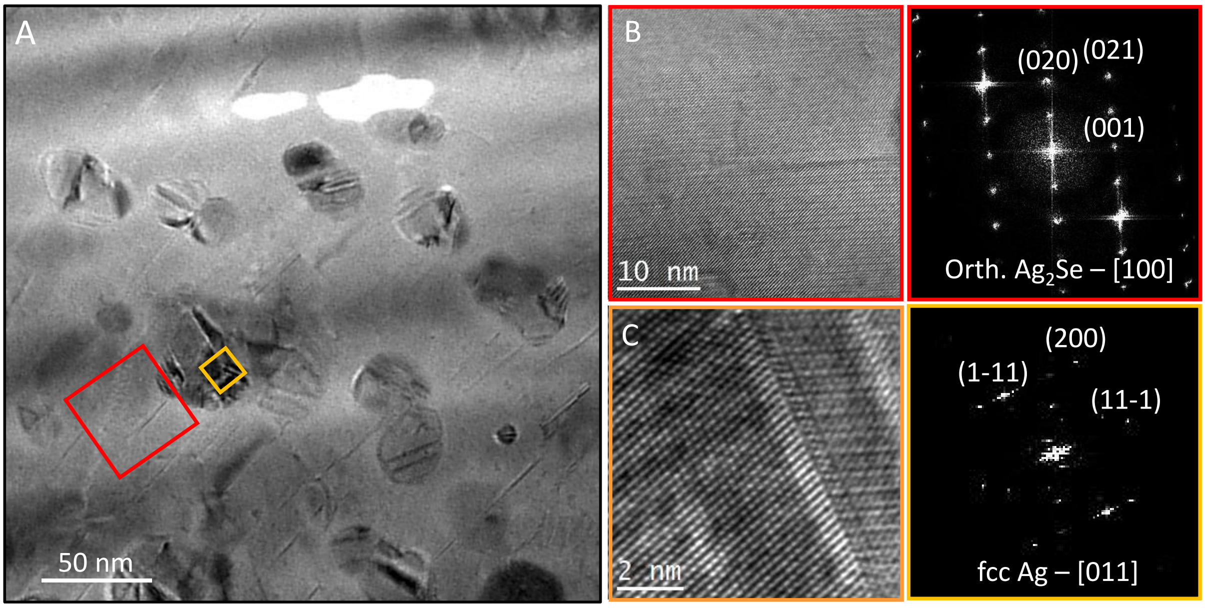

Figure S13. A) TEM image of the untreated Ag_2Se matrix with multiple nanoparticles distributed throughout. B) HRTEM image of the area marked by the red square with its corresponding FFT pattern outlined in red. C) HRTEM image of the area marked by the orange square with its corresponding FFT pattern outlined in orange.

Dense dislocations within the Ag2Se-7%CdSe pellets microstructure

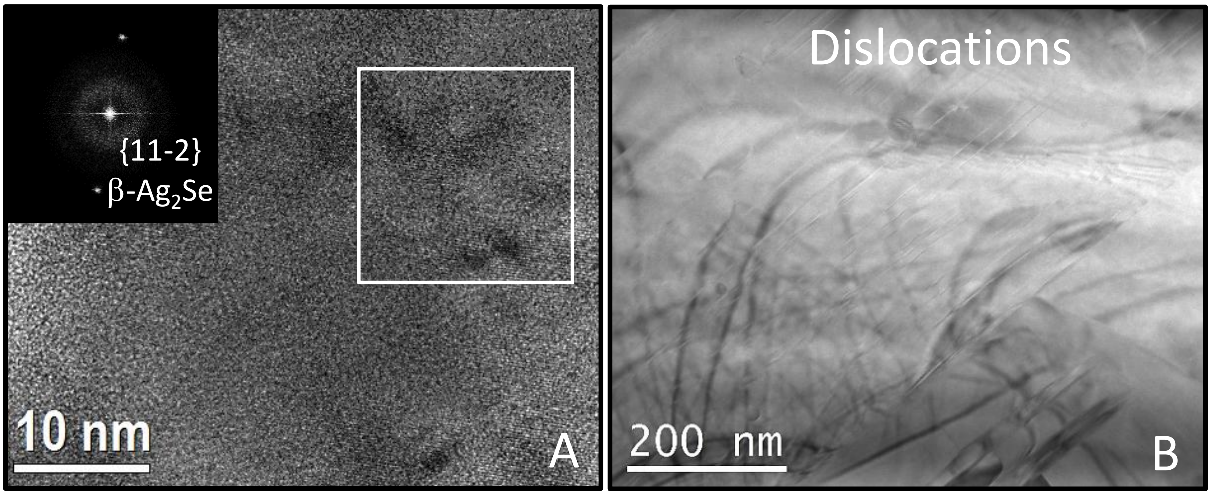

Figure S14. A) HRTEM: A HRTEM image of the Ag_2Se-7%CdSe NCP showcases the intricate structure of the Ag_2Se matrix. The inset reveals the FFT pattern that matches the HRTEM image of Ag_2Se. B) TEM image showcasing dislocations within the pellet's microstructure.

CdSe nanodomains within the Ag_2Se-7%CdSe pellet

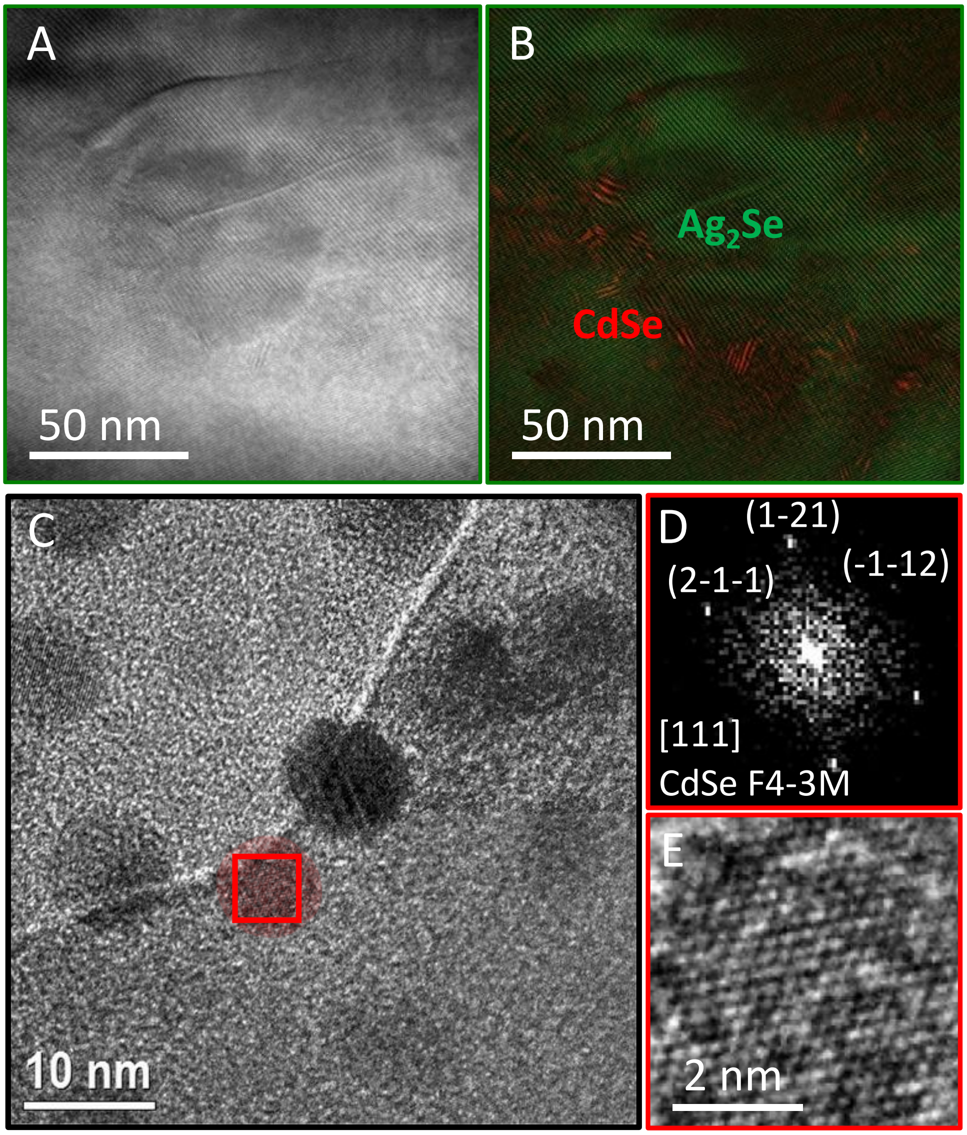

Figure S15. A) A HRTEM image showing the presence of small crystallites within the Ag_2Se matrix, B) FFT pattern of the entire region, revealing two distinctive features: a spot pattern generated by the Ag_2Se matrix grain (green) and a ring pattern generated by the CdSe nanocrystallites (red). Upon analysing over fifty CdSe crystallites, the average size of these CdSe nanodomains in this region has been determined to be 4.5 ± 1.2 nm. C-E) Another HRTEM image of the Ag_2Se-7%CdSe NCP, showing nanometer sized CdSe particles embedded within the Ag_2Se matrix. D) FFT pattern obtained from the region marked by the red square in (C), indexed to the [111] zone axis of cubic CdSe (space group F4-3M). E) Magnified view of the region highlighted in the red square in (C).

Transport data for different Ag:Se ratio and with/without en/edtH_2 treatment

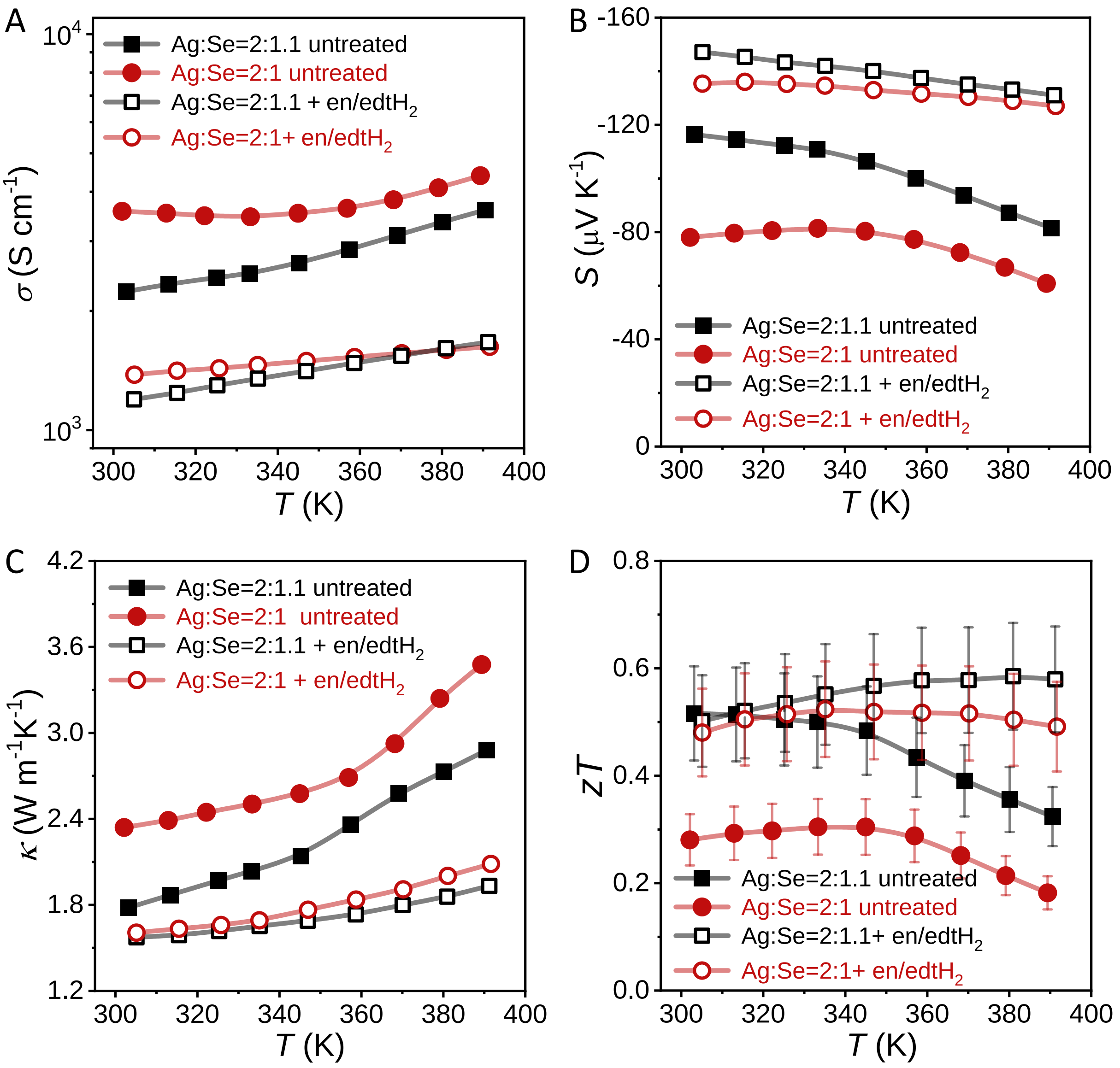

Figure S16. Evaluation of the TE properties of Ag_2Se pellets synthesized from Ag and Se precursors with molar ratios of 2:1 and 2:1.1, comparing untreated samples and those treated with en/edtH_2. A) electrical conductivity (*σ*); B) Seebeck coefficient (*S*); C) thermal conductivity (*κ*); and D) figure-of-merit (*zT*).

XRD pattern of Ag_2O powder and Ag-thiolates treated with CdSe complexes

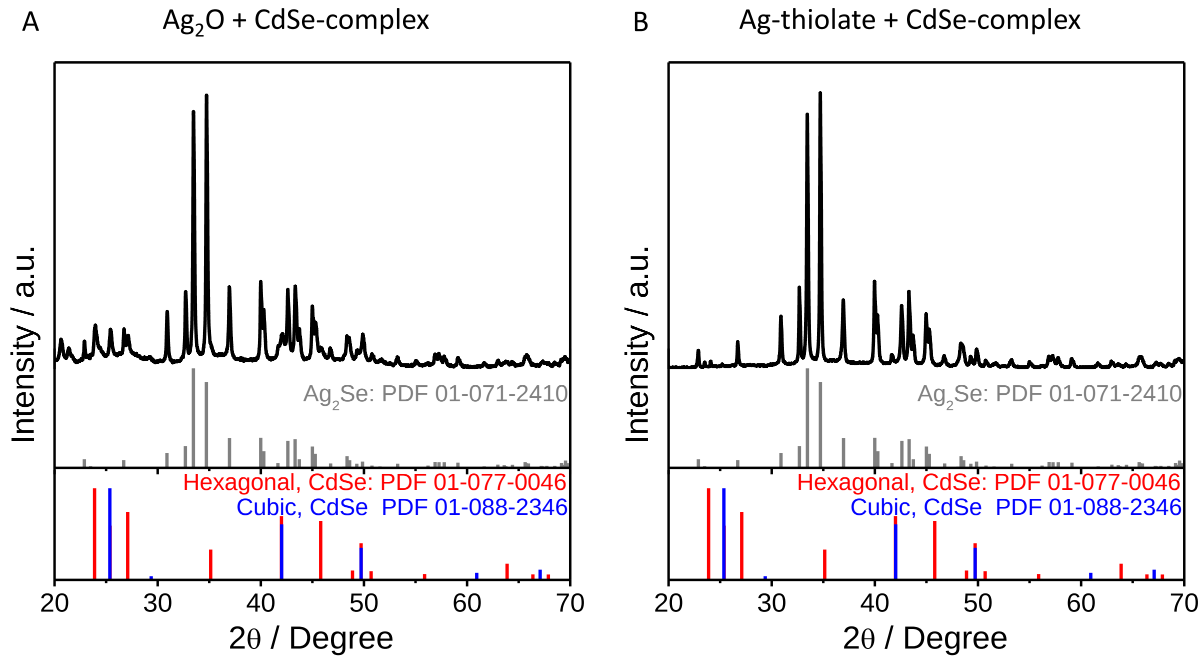

Figure S17. A) XRD pattern displaying Ag_2Se, with crystalline CdSe phase at room temperature. B) XRD pattern of a sample showing only crystalline Ag_2Se, with no detectable CdSe diffraction peaks. Standard diffraction references are included for orthorhombic Ag_2Se (PDF# 01-071-2410), hexagonal CdSe (PDF# 01-077-0046), and cubic CdSe (PDF# 01-088-2346).

In-situ TEM heating experiment showing CdSe redistribution with annealing

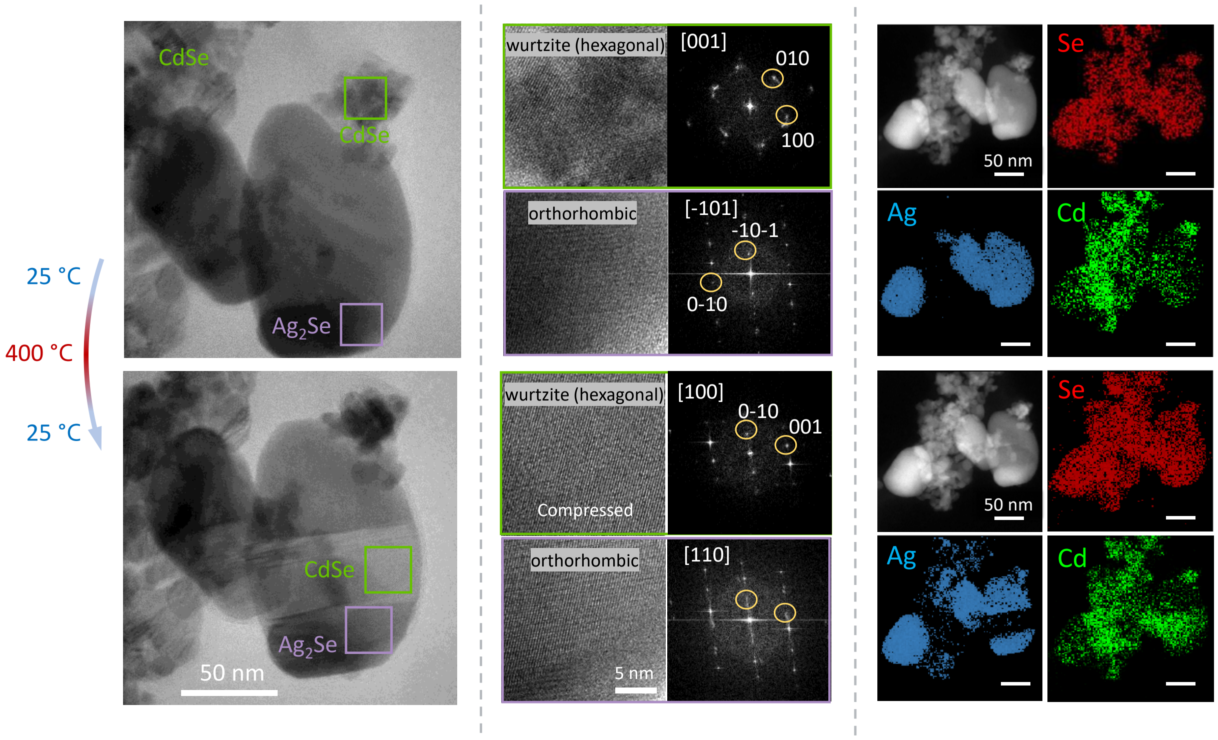

Figure S18. *In-situ* TEM heating experiment showing the presence of crystalline CdSe (wurtzite phase) already at room temperature, indicating that its formation occurs prior to thermal treatment. Upon heating, no evidence of Cd-Ag alloying is detected, consistent with the immiscibility of Cd and Ag in the investigated temperature range. Elemental mapping confirms the spatial separation of CdSe and Ag_2Se domains, with temperature-dependent redistribution of Cd and Ag.

Pellet density and composition

Table S1. Relative densities of Ag_2Se-x%CdSe (x=0, 2, 4, 6, 7 and 10) pellets obtained from absolute values measured with the Archimedes' method (*ca.* 2% error) compared with the theoretical value 8.22 g cm^{-3} of Ag_2Se.

Sample	x=0	x=2	x=4	x=6	x=7	x=10
Measured density (g/cm^3)	8.15	8.01	7.99	7.87	7.83	7.85
Relative density (%)	99.1	97.4	97.2	96.5	96.1	95.5

Table S2. Composition of Ag_2Se-x%CdSe (x=0, 2, 4, 6, 7 and 10) pellets obtained from ICP-OES (Related to Se=1).

Element atomic ratio	x=0	x=2	x=4	x=6	x=7	x=10
Ag	2.04	2.02	2	1.96	1.96	1.94
Se	1	1	1	1	1	1
Cd	0	0.02	0.03	0.05	0.07	0.09

Lattice parameters

Table S3. Comparison of lattice parameters of Ag_2Se-x%CdSe (x=0, 2, 4, 6, 7 and 10) samples obtained by Rietveld refinement (denoted as "*R*") and Bragg equation (denoted as "*B*") method.

Sample	Method	***a*** **(Å)**	***b*** **(Å)**	***c*** **(Å)**
x=0	*R*	4.3370	7.0651	7.7753
	B	4.3367	7.0683	7.7736
x=2	*R*	4.3397	7.0590	7.7675
	B	4.3340	7.0662	7.7722
x=4	*R*	4.3390	7.0575	7.7632
	B	4.3391	7.0601	7.7718
x=6	*R*	4.3401	7.0548	7.7618
	B	4.3399	7.0588	7.7693
x=7	*R*	4.3379	7.0500	7.7644
	B	4.3381	7.0574	7.7708
x=10	*R*	4.3369	7.0659	7.7690
	B	4.3384	7.0658	7.7730

Room-temperature hole concentration (n_H) and mobility (μ_H)

Table S4. Room-temperature Hall carrier concentration (n_H) and mobility (μ_H) of Ag_2Se-x%CdSe (x=0, 2, 4, 6, 7 and 10) NCPs. The values provided correspond to the average of 10 measurements and the estimated error was *ca.* 10%.

Sample	n_H ($\times 10^{18}$ cm^{-3})	μ_H ($\times 10^{2}$ cm^2 $V^{-1}s^{-1}$)
x=0	13.0	1160
x=2	11.5	980
x=4	10.6	837
x=6	9.7	800
x=7	9.0	760
x=10	8.2	650

Ag-thiolate complexes

Table S5. ESI-HRMS data of isolated silver thiolate complexes, re-dissolved in DMSO (1 mg/mL) and diluted with ACN: DMSO (1:1 vol: vol) to 0.1 mg/mL.

Putative molecular formula	Theoretical *m*/*z**	Measured *m*/*z**	Mass error (ppm)
$[AgS_2C_2H_4]^-$	198.8811	198.8806	-2.51
$[AgS_3C_2H_6]^-$	232.8688	232.8688	0.00
$[AgS_3C_4H_8]^-$	258.8845	258.8847	0.77
$[AgS_4C_6H_{12}]^-$	318.8878	318.8880	0.63
$[AgS_5C_8H_{16}]^-$	378.8901	378.8912	1.06
$[Ag_2S_4C_4H_9]^-$	398.7694	398.7697	0.75
$[AgS_6C_{10}H_{20}]^-$	438.8946	438.8953	1.59
$[AgS_8C_8H_{16}]^-$	474.8074	474.8081	1.47
$[Ag_2S_6C_6H_{13}]^-$	490.7449	490.7458	1.83
$[AgS_7C_{12}H_{24}]^-$	498.8979	498.8991	2.41
$[Ag_3S_4C_4H_8]^-$	504.6667	504.6677	1.98
$[Ag_2S_6C_8H_{17}]^-$	518.7762	518.7770	1.54
$[Ag_2S_7C_{10}H_{21}]^-$	578.7796	578.7804	1.38
$[Ag_2S_8C_{12}H_{25}]^-$	638.7829	638.7841	1.88
$[Ag_4S_5C_4H_9]^-$	644.5517	644.5527	1.55
$[Ag_2S_9C_{14}H_{29}]^-$	698.7863	698.7887	3.43
$[Ag_4S_6C_6H_{13}]^-$	704.5551	704.5570	2.70
$[Ag_4S_7C_8H_{17}]^-$	764.5584	764.5601	2.22
$[Ag_5S_6C_6H_{12}]^-$	810.4523	810.4544	2.59
$[Ag_4S_8C_{10}H_{21}]^-$	824.5618	824.5637	2.30
$[Ag_5S_7C_8H_{16}]^-$	870.4557	870.4584	3.10
$[Ag_4S_9C_{12}H_{25}]^-$	884.5652	884.5669	1.92

* Reported *m/z* corresponds to the monoisotopic peak

CdSe complexes

Table S6. ESI-HRMS data of CdSe molecular complexes prepared by dissolving CdO and Se powder in thiol-amine, isolating the complex, re-dissolving in DMSO (1 mg/mL) and diluting with ACN to 0.1 mg/mL.

Putative molecular formula	Theoretical *m/z**	Measured *m/z**	Mass error (ppm)
$[Se_3]^-$	237.7509	237.7503	-2.52
$[CdSe_6S_2]^{2-}$	326.6747	326.6747	0.00
$[CdSe_8]^{2-}$	372.6204	372.6210	1.61
$[CdSe_4]^-$	429.5717	429.5720	0.70
$[CdSe_4S]^-$	463.5426	463.5430	0.86
$[CdSe_5]^-$	509.4878	509.4877	-0.20
$[CdSe_5S]^-$	541.4599	541.4603	0.74
$[CdSe_6]^-$	589.4046	589.4053	1.19
$[CdSe_6S]^-$	621.3767	621.3775	1.29
$[CdSe_7]^-$	667.3224	667.3233	1.35

* Reported *m/z* corresponds to the highest peak in the measured isotopic envelope

Cd-thiolate complexes

Table S7. ESI-HRMS data of Cd-thiolate complexes prepared by reacting Ag-thiolates (prepared from Ag_2O) and CdSe complexes in MFA and isolating the resulting soluble complexes. The Cd-thiolate complexes are re-dissolved in DMSO (1 mg/mL) and diluted with ACN: DMSO (1:1 vol: vol) to 0.1 mg/mL.

**Putative molecular formula**	**Theoretical *m/z****	**Measured *m/z****	**Mass error (ppm)**
$[CdS_3C_2H_5]^-$	238.8592	238.8598	2.51
$[CdS_4C_4H_9]^-$	298.8626	298.8637	3.68
$[CdS_5C_6H_{13}]^-$	358.8659	358.8671	3.34
$[CdS_6C_8H_{17}]^-$	418.8691	418.8706	3.58
$[CdS_7C_{10}H_{21}]^-$	478.8723	478.8739	3.34
$[CdS_8C_{12}H_{25}]^-$	538.8755	538.8775	3.71
$[CdS_9C_{14}H_{29}]^-$	598.8795	598.8811	2.67
$[CdS_{10}C_{16}H_{33}]^-$	658.8820	658.8848	4.25
$[CdS_{11}C_{18}H_{37}]^-$	718.8853	718.8884	4.31
$[CdS_{12}C_{20}H_{41}]^-$	778.8885	778.8922	4.75
$[CdS1_3C_{22}H_{45}]^-$	838.8918	838.8956	4.53

* Reported *m/z* corresponds to the highest peak in the measured isotopic envelope

Theoretical calculation details

The single parabolic band (SPB) model was employed to calculate the Pisarenko curve and the n_H-dependent predicted zT values (Figure 3H) at a fixed temperature 300 K (dotted lines) and 380 K (solid lines), and the κ_L used in the calculations are derived from Figure 3E. Within the SPB model for the detailed calculations, the carrier transport property analysis was used as follows:[24,25]

The Seebeck coefficient,

$$S(\eta) = \frac{\kappa_B}{e}\left[\frac{(r+5/2)\cdot F_{r+3/2}(\eta)}{(r+3/2)\cdot F_{r+1/2}(\eta)} - \eta\right] \quad (S1)$$

The Hall carrier concentration,

$$n_H = \frac{1}{e\cdot R_H} = \frac{(2m^*\cdot\kappa_B T)^{3/2}}{3\pi^2\hbar^3}\cdot\frac{(r+3/2)^2\cdot F_{r+1/2}^2(\eta)}{(2r+3/2)\cdot F_{2r+1/2}(\eta)} \quad (S2)$$

The Hall mobility,

$$\mu_H = \left[\frac{e\pi\hbar^4}{\sqrt{2}(\kappa_B T)^{3/2}}\frac{C_l}{{E_{def}}^2(m^*)^{5/2}}\right]\frac{(2r+3/2)\cdot F_{2r+1/2}(\eta)}{(r+3/2)^2\cdot F_{r+1/2}(\eta)} \quad (S3)$$

Lorenz Factor,

$$L = \left(\frac{\kappa_B}{e}\right)^2\left\{\frac{(r+7/2)\cdot F_{r+5/2}(\eta)}{(r+3/2)\cdot F_{r+1/2}(\eta)} - \left[\frac{(r+5/2)\cdot F_{r+3/2}(\eta)}{(r+3/2)\cdot F_{r+1/2}(\eta)}\right]^2\right\} \quad (S4)$$

Where

$$F_x(\eta) = \int_0^\infty \frac{\varepsilon^x}{1+e^{(\varepsilon-\eta)}}d\varepsilon \quad (S5)$$

is the Fermi integral.

In the above equations, S, μ_H, η, κ_B, e, r, R_H, $\hbar$, C_l, E_{def}, m^*, and L are the Seebeck coefficient, the carrier mobility, the reduced Fermi level, the Boltzmann constant, the electron charge, the carrier scattering factor (r = -1/2 for acoustic phonon scattering), the Hall coefficient, the reduced plank

constant, the elastic constant for longitudinal vibrations, the deformation potential coefficient, the density of state effective mass, and the Lorenz number, respectively.

Weight mobility and Phonon density of states (PDOS) calculations

Weighted mobilities (Figure S9C) were calculated from electrical conductivity (σ) and Seebeck coefficient (S) measurements according to the equation:[26]

$$\mu_W = \frac{3h^3\sigma}{8\pi e(2m_e k_B T)^{3/2}}\left[\frac{exp\left[\frac{e|S|}{k_B}-2\right]}{1+exp\left[\frac{-5e|S|}{k_B}+5\right]}+\frac{\frac{3}{\pi^2}\frac{e|S|}{k_B}}{1+exp\left[\frac{5e|S|}{k_B}-5\right]}\right] \quad (S6)$$

In the above equation σ, S, h, κ_B, e and m_e, are the electrical conductivity, Seebeck coefficient, Plank's constant, Boltzmann's constant, electron charge and electrons mass.

The calculation of the phonon dispersion and group velocity (Figure 3A-C) in the main text were carried out using the Phonopy package,[27,28] and the force constant matrix was generated using the finite displacement method, with a 2 × 2 × 2 supercell.

Quality factor calculation

The quality factor (B) was calculated using the following equation [29,30]:

$$B = 9\frac{\mu_W}{\kappa_L}\left(\frac{T}{300}\right)^{5/2} \quad (S7)$$

In the above equation μ_w, T and κ_L, are the weight mobility, absolute temperature, and lattice thermal conductivity, respectively.

References

(1) Wang, H.; Liu, X.; Zhang, B.; Huang, L.; Yang, M.; Zhang, X.; Zhang, H.; Wang, G.; Zhou, X.; Han, G. General Surfactant-Free Synthesis of Binary Silver Chalcogenides with Tuneable Thermoelectric Properties. *Chem. Eng. J.* **2020**, *393*, 124763.

(2) McCarthy, C. L.; Webber, D. H.; Schueller, E. C.; Brutchey, R. L. Solution-Phase Conversion of Bulk Metal Oxides to Metal Chalcogenides Using a Simple Thiol-Amine Solvent Mixture. *Angew. Chem. Int. Ed.* **2015**, *54* (29), 8378–8381.

(3) Liu, Y.; Calcabrini, M.; Yu, Y.; Lee, S.; Chang, C.; David, J.; Ghosh, T.; Spadaro, M. C.; Xie, C.; Cojocaru-Mirédin, O.; Arbiol, J.; Ibáñez, M. Defect Engineering in Solution-Processed Polycrystalline SnSe Leads to High Thermoelectric Performance. *ACS Nano* **2022**, *16* (1), 78–88.

(4) Jood, P.; Chetty, R.; Ohta, M. Structural Stability Enables High Thermoelectric Performance in Room Temperature Ag_2Se. *J. Mater. Chem. A* **2020**, *8* (26), 13024–13037.

(5) Yang, D.; Shi, X.-L.; Li, M.; Nisar, M.; Mansoor, A.; Chen, S.; Chen, Y.; Li, F.; Ma, H.; Liang, G. X.; Zhang, X.; Liu, W.; Fan, P.; Zheng, Z.; Chen, Z.-G. Flexible Power Generators by Ag_2Se Thin Films with Record-High Thermoelectric Performance. *Nat. Commun.* **2024**, *15* (1), 923.

(6) Liang, J.; Qiu, P.; Zhu, Y.; Huang, H.; Gao, Z.; Zhang, Z.; Shi, X.; Chen, L. Crystalline Structure-Dependent Mechanical and Thermoelectric Performance in $Ag_2Se_{1-x}S_x$ System. *Research* **2020**, *2020*, 6591981.

(7) Feng, B.; Cheng, Y.; Liu, C.; Gao, J.; Wu, G.; Bai, X.; Si, R.; Li, W.; Guo, Y.; Miao, L. Ag Interstitial Inhibition and Phonon Scattering at the ZnSe Nano-Precipitates to Enhance the Thermoelectric Performance of Ag_2Se. *ACS Appl. Energy Mater.* **2023**, *6* (5), 2804–2811.

(8) Nan, B.; Li, M.; Zhang, Y.; Xiao, K.; Lim, K. H.; Chang, C.; Han, X.; Zuo, Y.; Li, J.; Arbiol, J.; Llorca, J.; Ibáñez, M.; Cabot, A. Engineering of Thermoelectric Composites Based on Silver Selenide in Aqueous Solution and Ambient Temperature. *ACS Appl. Electron. Mater.* **2024**, *6* (5), 2807–2815.

(9) Zheng, Z.-H.; Li, Y.-L.; Niu, J.-Y.; Wei, M.; Zhang, D.-L.; Zhong, Y.; Nisar, M.; Abbas, A.; Chen, S.; Li, F. Significantly (00l)-Textured Ag_2Se Thin Films with Excellent Thermoelectric Performance for Flexible Power Applications. *J. Mater. Chem. A* **2022**, *10* (40), 21603–21610.

(10) Li, D.; Zhang, J. H.; Li, J. M.; Zhang, J.; Qin, X. Y. High Thermoelectric Performance for an Ag_2Se-Based Material Prepared by a Wet Chemical Method. *Mater. Chem. Front.* **2020**, *4* (3), 875–880.

(11) Chen, J.; Sun, Q.; Bao, D.; Liu, T.; Liu, W.-D.; Liu, C.; Tang, J.; Zhou, D.; Yang, L.; Chen, Z.-G. Hierarchical Structures Advance Thermoelectric Properties of Porous n-Type β-Ag_2Se. *ACS Appl. Mater. Interfaces* **2020**, *12* (46), 51523–51529.

(12) Li, D.; Zhang, B. L.; Ming, H. W.; Wang, L.; Zu, Y.; Qin, X. Y. Liquid-Phase Manipulation Securing Enhanced Thermoelectric Performance of Ag_2Se. *ACS Appl. Mater. Interfaces* **2021**, *13* (29), 34543–34549.

(13) Wang, P.; Chen, J.-L.; Zhou, Q.; Liao, Y. T.; Peng, Y.; Liang, J. S.; Miao, L. Enhancing the Thermoelectric Performance of Ag_2Se by Non-Stoichiometric Defects. *Appl. Phys. Lett.* **2022**, *120* (19), 193902.

(14) Yang, D.; Su, X.; Meng, F.; Wang, S.; Yan, Y.; Yang, J.; He, J.; Zhang, Q.; Uher, C.; Kanatzidis, M. G. Facile Room Temperature Solventless Synthesis of High Thermoelectric Performance Ag_2Se via a Dissociative Adsorption Reaction. *J. Mater. Chem. A* **2017**, *5* (44), 23243–23251.

(15) Lim, K. H.; Xia, Y.; Xu, L.; Zhao, M.; Li, M.; Cheng, Y.; Mao, J.; Wang, S.; Chen, L.; Tsang, S. W.; Liu, P.; Wang, Q.; Yang, X.; Wang, W.-J.; Cabot, A.; Hong, M.; Zhang, Y.; Liu, Y. Thermoelectric Performance in Ag_2Se Nanocomposites: The Role of Interstitial Ag and Pb Orbital Hybridization. *Chem. Eng. J.* **2025**, *511*, 162265.

(16) Kleinhanns, T.; Milillo, F.; Calcabrini, M.; Fiedler, C.; Horta, S.; Balazs, D.; Strumolo, M. J.; Hasler, R.; Llorca, J.; Tkadletz, M.; Brutchey, R. L.; Ibáñez, M. A Route to High Thermoelectric Performance: Solution-Based Control of Microstructure and Composition in Ag_2Se. *Adv. Energy Mater.* **2024**, *14* (22), 2400408.

(17) Wang, H.; Han, G.; Zhang, B.; Chen, Y.; Liu, X.; Zhang, K.; Lu, X.; Wang, G.; Zhou, X. $AgSbSe_2$ Inclusions Enabling High Thermoelectric and Mechanical Performance in n-Type Ag_2Se -Based Composites. *Acta Mater.* **2023**, *248*, 118753.

(18) Wang, H.; Liu, X.; Zhou, Z.; Wu, H.; Chen, Y.; Zhang, B.; Wang, G.; Zhou, X.; Han, G. Constructing n-Type Ag_2Se/CNTs Composites toward Synergistically Enhanced Thermoelectric and Mechanical Performance. *Acta Mater.* **2022**, *223*, 117502.

(19) Chen, J.; Sun, Q.; Bao, D.; Tian, B.-Z.; Wang, Z.; Tang, J.; Zhou, D.; Yang, L.; Chen, Z.-G. Simultaneously Enhanced Strength and Plasticity of Ag_2Se-Based Thermoelectric Materials Endowed by Nano-Twinned CuAgSe Secondary Phase. *Acta Mater.* **2021**, *220*, 117335.

(20) Lim, K. H.; Wong, K. W.; Liu, Y.; Zhang, Y.; Cadavid, D.; Cabot, A.; Ng, K. M. Critical Role of Nanoinclusions in Silver Selenide Nanocomposites as a Promising Room Temperature Thermoelectric Material. *J. Mater. Chem. C* **2019**, *7* (9), 2646-2652.

(21) Ahmad, S.; Sarkar, P.; Bhatt, P.; Bhattacharya, S.; Navaneethan, M.; Basu, R.; Bhatt, R.; Bohra, A.; Debnath, A. K.; Muthe, K. P. Improved Thermoelectric Performance of $Ag_{2-x}Al_xSe$ through Formation of AgAl Phase. *Appl. Phys. Lett.* **2022**, *121* (17), 173905.

(22) Chen, J.; Yuan, H.; Zhu, Y.-K.; Zheng, K.; Ge, Z.-H.; Tang, J.; Zhou, D.; Yang, L.; Chen, Z.-G. Ternary $Ag_2Se_{1-x}Te_x$: A Near-Room-Temperature Thermoelectric Material with a Potentially High Figure of Merit. *Inorg. Chem.* **2021**, *60* (18), 14165–14173.

(23) Tee, S. Y.; Tan, X. Y.; Wang, X.; Lee, C. J. J.; Win, K. Y.; Ni, X. P.; Teo, S. L.; Seng, D. H. L.; Tanaka, Y.; Han, M.-Y. Aqueous Synthesis, Doping, and Processing of n-Type Ag_2Se for High Thermoelectric Performance at near-Room-Temperature. *Inorg. Chem.* **2022**, *61* (17), 6451–6458.

(24) Shen, J.; Chen, Z.; Lin, S.; Zheng, L.; Li, W.; Pei, Y. Single Parabolic Band Behavior of Thermoelectric p-Type $CuGaTe_2$. *J. Mater. Chem. C* **2015**, *4* (1), 209–214.

(25) Goldsmid, H. J. Introduction to Thermoelectricity; Springer, 2010; Vol. 121.

(26) Snyder, G. J.; Snyder, A. H.; Wood, M.; Gurunathan, R.; Snyder, B. H.; Niu, C. Weighted Mobility. *Adv. Mater.* **2020**, *32* (25), 2001537.

(27) Togo, A. First-Principles Phonon Calculations with Phonopy and Phono3py. *J. Phys. Soc. Japan* **2023**, *92* (1), 12001.

(28) Togo, A.; Chaput, L.; Tadano, T.; Tanaka, I. Implementation Strategies in Phonopy and Phono3py. *J. Phys. Condens. Matter* **2023**, *35* (35), 353001.

(29) Qin, B.; He, W.; Zhao, L.-D. Estimation of the Potential Performance in p-Type SnSe Crystals through Evaluating Weighted Mobility and Effective Mass. *J. Mater.* **2020**, *6* (4), 671–676.

(30) Li, M.; Liu, Y.; Zhang, Y.; Han, X.; Xiao, K.; Nabahat, M.; Arbiol, J.; Llorca, J.; Ibañez, M.; Cabot, A. PbS–Pb–Cu_xS Composites for Thermoelectric Application. *ACS Appl. Mater. Interfaces* **2021**, *13* (43), 51373–51382.
